# Efficient high-throughput sequencing of a laser microdissected chromosome arm

**DOI:** 10.1186/1471-2164-14-357

**Published:** 2013-05-28

**Authors:** Eva Seifertova, Lyle B Zimmerman, Michael J Gilchrist, Jaroslav Macha, Svatava Kubickova, Halina Cernohorska, Vojtech Zarsky, Nick DL Owens, Abdul K Sesay, Tereza Tlapakova, Vladimir Krylov

**Affiliations:** 1 Charles University in Prague, Faculty of Science, Vinicna 7, Prague 2, 128 44, Czech Republic; 2 MRC National Institute for Medical Research, The Ridgeway, Mill Hill, London, NW7 1AA, UK; 3 Veterinary Research Institute, Hudcova 70, Brno, 621 00, Czech Republic

**Keywords:** *Xenopus*, *Tropicalis*, Chromosomes, Next generation sequencing, WGA, Genetic map

## Abstract

**Background:**

Genomic sequence assemblies are key tools for a broad range of gene function and evolutionary studies. The diploid amphibian *Xenopus tropicalis* plays a pivotal role in these fields due to its combination of experimental flexibility, diploid genome, and early-branching tetrapod taxonomic position, having diverged from the amniote lineage ~360 million years ago. A genome assembly and a genetic linkage map have recently been made available. Unfortunately, large gaps in the linkage map attenuate long-range integrity of the genome assembly.

**Results:**

We laser dissected the short arm of *X. tropicalis* chromosome 7 for next generation sequencing and computational mapping to the reference genome. This arm is of particular interest as it encodes the sex determination locus, but its genetic map contains large gaps which undermine available genome assemblies. Whole genome amplification of 15 laser-microdissected 7p arms followed by next generation sequencing yielded ~35 million reads, over four million of which uniquely mapped to the *X. tropicalis* genome. Our analysis placed more than 200 previously unmapped scaffolds on the analyzed chromosome arm, providing valuable low-resolution physical map information for *de novo* genome assembly.

**Conclusion:**

We present a new approach for improving and validating genetic maps and sequence assemblies. Whole genome amplification of 15 microdissected chromosome arms provided sufficient high-quality material for localizing previously unmapped scaffolds and genes as well as recognizing mislocalized scaffolds.

## Background

Recently, complete genomes of many important model organisms have been assembled using either Sanger or next generation sequencing such as Solexa (Illumina), Roche 454, SOLiD etc. [1]. However, repetitive elements in higher eukaryotic genomes interfere with assembly of sequence information alone into unified chromosome-scale scaffolds [2]. This obstacle is usually overcome by construction of physical or meiotic linkage maps to provide long-range contiguity. Physical mapping can be accomplished by a variety of methods including restriction analysis of BAC libraries, radiation hybrid panels, and direct visualization of marker positions on chromosomes using fluorescent *in situ* hybridization (FISH). The latter approach is quite accurate, but only a few markers can be localized in one run. In meiotic linkage mapping, relationships among polymorphic marker sequences are determined by relative frequency of recombination. However, recombination frequency is highly variable, often decreasing near centromeres and high in hotspots, making it difficult to compare genetic and physical distances. In addition, resolution of linkage analysis depends on the type of markers chosen and their abundance.

The diploid amphibian *Xenopus tropicalis* plays a key role in basic biological research. This model system is particularly valuable for studies of early vertebrate embryonic development [3,4], functional genomics [5,6], cell biology [3,7], and vertebrate genome evolution [8]. Its 1.7 × 10^9^ bp genome was sequenced [9] and a genetic map covering its 10 chromosomes was constructed [10]. Two genome assemblies are in wide use, both available on http://www.xenbase.org. The version 4.1 assembly (v4.1, Joint Genome Institute) is solely sequence-based and consists of 19,501 scaffolds. A more recent assembly, version 7.1 (v7.1, [9], discussed in [11]), orders reassembled scaffolds using meiotic map and synteny information into a ‘main assembly’ of 10 chromosome-scale superscaffolds covering ~75% of the genome, with another ~7000 small ‘orphan’ scaffolds not incorporated into the main assembly. While this long-range assembly is extremely useful, regions assembled by inferring shared gene order with more complete amniote assemblies must be considered provisional, as synteny is not always conserved over large phylogenetic distances. Likewise, the genetic map only locates v4.1 scaffolds covering ~62% of the *X. tropicalis* genome, or about 758 of ~1300 v4.1 [10] scaffolds larger than 100 kb; polymorphic markers were not obtained for the remaining ‘unmapped scaffolds’. The largest gaps in the genetic map include the entire short arm of chromosome 2, and a ~15 cM span inside the distalmost marker on the p arm of chromosome 7. Interestingly, the gap on chromosome 7 appears to contain the *X. tropicalis* sex determining locus [12], although an independent marker analysis suggests that there is not a large region of sex-specific sequence [13] which might interfere with meiotic mapping.

**Figure 1:**
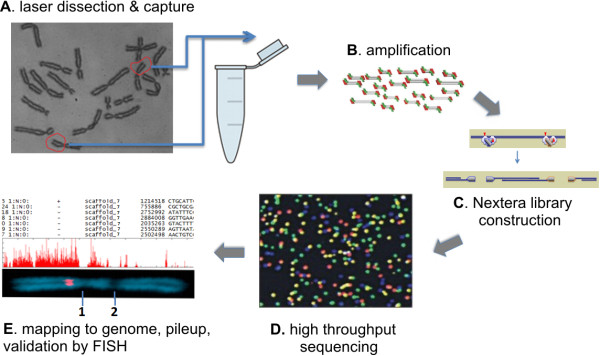
**Chromosome dissection and sequencing workflow. (A)** Dissociated *Xenopus tropicalis* froglet testes were cultured in colchicine, and 15 Chromosome 7 short arms were laser-dissected, collected and **(B)** amplified using Sigma WGA3/WGA4 systems. **(C)** Sequencing libraries were prepared by Nextera transposome-mediated simultaneous fragmentation and adaptor ligation to minimize resequencing WGA adaptors, and **(D)** sequenced on an Illumina GAII. **(E)** The resulting reads were trimmed and mapped to *X. tropicalis* genome assemblies v4.1 and v7.1 using Bowtie, visualized on scaffolds/chromosomes, and selected positions were validated by FISH-TSA. 1- Centromere, 2- secondary constriction.

To identify sequences within these gaps as well as map and assembly errors, we developed an improved method based on high-throughput sequencing of laser microdissected chromosome arms (Figure 1). Recent technical advances have enabled low cost genome sequencing of nearly any species [14,15]. However, direct sequencing of specific chromosomes or chromosomal regions has only been successful in species where individual chromosomes could be separated by flow sorting (reviewed in [16]). Microdissection of chromosomes has been attempted, but this approach depends on whole-genome amplification due to practical limits on the amount of starting material [17]. In the only published study, sequence resolution was low, probably due to the poor yield and quality of the DNA obtained [18].

Here we used 15 microdissected copies of the short arm of chromosome 7. This small amount of material was then subjected to whole genome amplification (WGA), and sequencing libraries were constructed by transposase-based simultaneous fragmentation and primer insertion, and then sequenced. For WGA, we chose the Sigma GenomePlex single cell kit since it amplified more markers and yielded the highest quantity of DNA relative to other systems [19], and has been successfully used to amplify microdissected human chromosomes for SNP genotyping [17]. This combination provided excellent read depth for placing previously unmapped scaffolds and genes to the 7p region, as well as recognizing mislocalized scaffolds.

## Results

We prepared metaphase chromosomes from primary cell cultures of dissected subadult frog testes [19]. We then laser microdissected 15 copies of the short arm of chromosome 7, which is easily distinguishable due to a secondary constriction in its q arm (Figure 1E). To ensure harvest of the entire p arm, the laser path was targeted between the constriction and centromere (Figure 1E). The extremely small amount of starting material was then amplified by WGA, with a total yield of ~20 μg averaging 500–600 bp in size. For library construction, we wished to minimize resequencing WGA primers added to ends of genomic fragments. To that end, we used a transposase-based simultaneous fragmentation/adaptor ligation method (Nextera, Illumina Inc.) where sequencing primer insertions are biased away from DNA ends. 80 bp reads were then obtained in a single lane of Illumina GAII. Reads were mapped to both versions of *Xenopus tropicalis* assemblies (v4.1 and v7.1) using Bowtie.

### Comparison to v4.1 assembly

**Figure 2:**
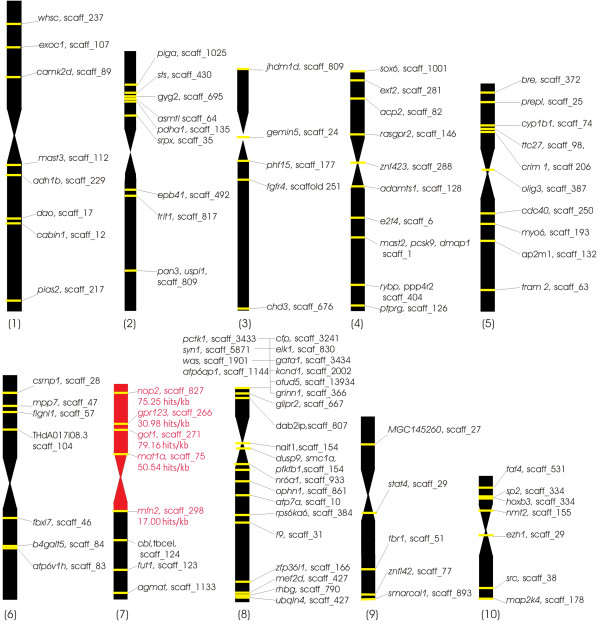
**Uniquely-localized read distribution on physically mapped scaffolds.** Schematic of *X. tropicalis* karyotype showing genes mapped by FISH-TSA [8,10,20] and cognate v4.1 scaffolds (scaff_number). High read density (17–80 hits/kb) is seen for scaffolds localized to the microdissected region (red region). Genes that physically localized to non-7p regions were all contained by scaffolds with <1 hit/kb (black regions).

In total, we obtained 35 million 80 bp reads, 18% of which mapped with a maximum of 2/80 mismatches to at least one location in the v4.1 genome assembly. Of these, 3,900,340 (11% of total) mapped to unique sites in the genome (‘hits’, shown in Additional file 1). Despite our efforts to minimize resequencing WGA primers, 30% of non-mapping reads contained at least 11 bases of either Illumina primer or the proprietary Sigma WGA sequence. The remaining non-mapping reads could be either contaminated by shorter stretches of primer or reflected misassembled or missing regions of the genome. Of the uniquely-mapped reads, nearly 70% mapped to scaffolds previously localized to the p arm of chromosome 7 by the genetic map [10], with the majority of those scaffolds showing high read/kb values (see Additional file 2). Since the published genetic map contains large gaps on chromosome 7, chromosome 2 and elsewhere, we also collated available cytogenetic map data from Fluorescence In Situ Hybridization with Tyramide Signal Amplification (FISH-TSA) analysis using cDNA probes [8,10,20], and evaluated read density on scaffolds anchored by known physical location of probes. Read density was congruent with the locations of all 90 physically-mapped scaffolds, including 4 markers in the 7p region (Figure 2, Additional file 3). Near the centromere (genetically mapped to 69 cM +/− 1 cM [12]), this ratio drops from approximately 70 to 17 uniquely-mapped reads/kb, probably due to increased repeat density diluting unique sequence in centromeric scaffolds. We conservatively used the hit ratio of 17 unique reads/kb observed for Scaffold_298, localized by FISH to just under the centromere [10], as the actual border giving the lowest acceptable hit/kb threshold. All scaffolds with lower than 17 hits/kb were considered false positives (2164 scaffolds containing 6.3% of hits, Table 1). The area between 72 and 73 cM contains scaffolds with around 1 read/kb indicating the laser cutting path. Below this borderline the hit/kb ratio rapidly falls more than 100×.

**Table 1:** **Reads locating uniquely to *Xenopus tropicalis *meiotically-mapped v4.1 scaffolds**

**All hits (%)**	**Hits to 7p v4.1 scaffolds (%)**	**Hits to above threshold non-7p v4.1 scaffolds (%)**	**Hits to above threshold unmapped scaffolds (%)**	**Hits to under-threshold scaffolds (%)**
3,900,340(100%)	2,638,303	135,487 (3.47%)	880,547	24,6003
	(67.64%)		(22.58%)	(6.3%)

Threshold is set to17 hit/kb (hit/kb of bordering scaffold_298).

3.47% of unique hits (135,487) were found in three scaffolds localized by the meiotic linkage map to non-7p regions. Two of these scaffolds were placed on the q arm of chromosome 7 in the linkage map despite relatively high read/kb values in our analysis (scaffold_598, 74.32 cM), 64 hits/kb and scaffold_1153, 96 cM, 57 hits/kb). The third scaffold (scaffold_302) mapped to the q arm of chromosome 8 (38.54 cM, 59 reads/kb). Since the high hit/kb ratios of these scaffolds are similar to those previously mapped to 7p region by FISH-TSA (scaffold_827, 75 reads/kb; scaffold_266, 31 reads/kb; scaffold_271, 79 reads/kb; and scaffold_75, 50 reads/kb) [10], we hypothesize that these sequences should be reassigned to the laser microdissected 7p arm. In total, we identified 231 v4.1 scaffolds not represented on the genetic map (22.5% of unique hits) with a read/kb value higher than 17 (border scaffold_298) which we can assign to the 7p region. 29 of these unmapped scaffolds are larger than 100 kb. On the other hand, 13 scaffolds with markers on 7p in the genetic map bore lower read/kb values than the threshold defined above, more consistent with a non-chromosome 7p location.

### Identification of hybrid scaffolds

**Figure 3:**
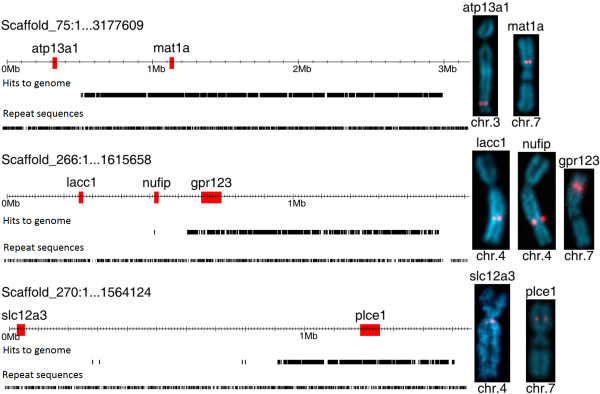
**FISH-TSA analysis of three hybrid scaffolds.** Selected genes from read-rich and read-absent areas of v4.1 scaffolds_75, _266, and _270 were physically mapped to *X. tropicalis* chromosomes using specific cDNA probes. Top line shows probe gene location on scaffold, second line shows distribution of uniquely-mapping reads, third line shows distribution of repetitive sequence (similar in hit-rich and hit-absent areas).

To identify misassembled (hybrid or broken) scaffolds, uniquely mapped reads were visualized on the *X. tropicalis* 4.1 assembly (Additional file 4). If a whole scaffold were present in the microdissected chromosome part, its full length should be covered by reads. Hybrid scaffolds may show gaps without any chromosome-specific hits, consistent with these sequences deriving from other chromosomal regions. We found that approximately 15% of scaffolds with meiotic map markers on 7p contained gaps in read coverage larger than 100 kb. These gaps in unique read hits are not generally caused by increased repeat density, which is similar in hit-rich and hit-absent areas (Figure 3), and the gaps usually contain genes, also suggesting that they are not due to repetitive sequence. FISH-TSA analysis of three suspected hybrid scaffolds (75, 266 and 270) confirmed assembly discrepancies, with probes from ‘hit rich’ regions localized to 7p, but cDNA probes from ‘hit absent’ areas decorating chromosomes 3 and 4 (Figure 3). Meiotic map markers from these scaffolds are also present in 7p and non-7p regions, confirming that these are hybrid ‘broken’ scaffolds rather than mapping errors. Our analysis thus helps pinpoint bad sequence joins in the scaffold assembly.

### Comparison to v7.1 assembly

**Figure 4:**
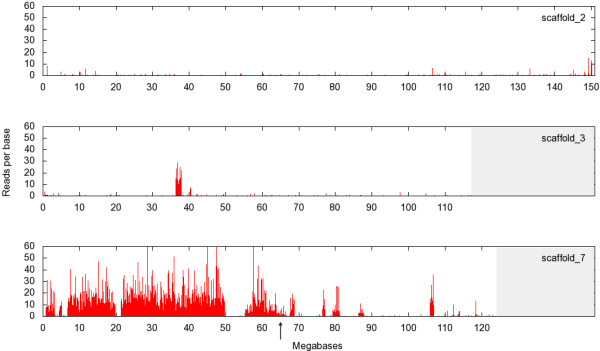
**Sequence of dissected chromosome arm identifies misassembled regions.** Pileup of uniquely-mapping reads from dissected chromosome 7p on *X. tropicalis* v7.1 assembly superscaffolds/chromosomes 2 (top), 3 (middle), and 7 (bottom). Reads mapping uniquely to typical non-7p regions exemplified by chromosomes 2 and 3 are rare, with the exception of a misassembled region at 37–38 Mb on chromosome 3. Most of the short arm (left side) of chromosome 7 is heavily decorated by reads, but gaps to the left of centromere (arrow) and read-dense regions to the right identify misassembled regions.

The v7.1 assembly incorporates both sequence information, long-range contiguity from the meiotic map, and gene synteny relationships from amniote genomes. ~75% of the coverage has been ordered provisionally into 10 large superscaffolds corresponding to the 10 *X. tropicalis* chromosomes. In our analysis, 4,489,728 reads placed uniquely on the v7.1 assembly (see Additional file 5). 80.8% of uniquely-mapping reads localized to scaffold (chromosome) 7, with 91% of these in the 0–60 Mb area roughly corresponding to the 7p region (Figure 4) with an average hit/kb ratio of 29.23. We defined this value (29 reads/kb) as a threshold for hit-positive v7.1 scaffolds. Five large gaps with much lower read density were observed between 0–0.7 Mb, 3.2–4.5 Mb, 5.2–6.7 Mb, 19.7–21.3 Mb and 49.7–55.2 Mb, consistent with areas that were misassembled in v7.1. The 60–65 Mb area is the approximate location of the presumptive laser path, but misassembly in this region is also possible. In the remaining part of scaffold 7 corresponding to 7q (65–120 Mb), we identified four large read-dense regions (67.7–68.8 Mb, 32 hits/kb; 76.5–77.0 Mb, 54 hits/kb; 79.3–81.0 Mb, 17 hits/kb; and 106.0–107.0 Mb, 57 hits/kb) which are likely to be located on the other arm of chromosome 7. Similar sharply-demarcated candidate 7p areas with high read/kb ratios were found also in chromosome/scaffolds 1 (114.0–115 Mb, 45 hits/kb), 3 (36.7–38.0 Mb, 62. hits/kb, see Figure 4) and 4 (11.0–11.5 Mb, 39 hits/kb, 14.5–15.35 Mb, 34. hits/kb) (shown in Additional file 6). Analysis of ‘orphan’ scaffolds not incorporated into the main chromosomal assembly identified another 14 orphan scaffolds larger than 100 kb showing high hit/kb values comparable to scaffold 7. Of these, scaffold 35 (27 hit/kb) is covered by high-density reads only on 30% of its length, consistent with a bad sequence join.

## Discussion

We present an improved technique for next generation sequencing of laser microdissected chromosome arms. Comparing a previous study [18] using 454 sequencing of DOP-PCR amplified human chromosome arms which yielded similar 80 bp average reads, we obtained approximately 30,000× more reads and 10,000× more unique hits to genome. The increase in usable reads allowed us to set high stringency conditions for mapping (97.5% identity).

As mentioned above, a large portion of the reads that did not map to the genome contained WGA primer sequences, despite precautions to minimize sequencing end fragments by the use of the Nextera system for building the sequencing library. Further optimization of library construction to avoid ends could increase usable read yield considerably.

The yield of mapped reads was comparable to that of a study where wheat flow-sorted chromosome arms were sequenced [21]. The flow sorting approach can collect abundant chromosomal DNA, but can only distinguish a minority of chromosomes in a given karyotype based on size alone [16]. Laser microdissection enables visual control and much greater discrimination among similarly-sized chromosomes, for example sorting by p/q arm ratio in metaphase spreads or using banding techniques. We verified our analysis using available FISH data for 93 relevant v4.1 scaffolds [8,10,20]. All known 7p scaffolds have a high unique read/kb ratio, whereas non-7p scaffolds show a maximum of 1 hit/kb without exceptions (see Figure 2 and Additional file 3). The total fraction of above-threshold reads mapping to chromosome 7p was 93.7% in the v4.1 assembly and 91% in v7.1, comparable to an analysis of human chromosome 19 [22] where 93% of reads mapped to the cognate region.

As mentioned above, the chromosome 7 linkage map contains a large gap between the most distal marker (0 cM) and the next one at 15 cM. We identified 264 scaffolds from the v4.1 assembly in 7p, only 49 of which are represented in the current linkage map. The remaining 215 scaffolds, with a total of size 17 Mb, had either unknown or incorrect positions. Many of these newly localized scaffolds are likely to be contained in the distal gap as well as smaller gaps elsewhere on the 7p linkage map.

Although v4.1 7p scaffolds show unique read/kb ratios ranging from 17 to 527, these values are sufficient to assign scaffolds to the laser microdissected area. Identification of scaffolds as hybrid or misassembled by virtue of unequal hit coverage was verified by FISH-TSA. Scaffolds close to the laser cut have a lower coverage (17–20 hits/kb), but these hits are evenly distributed across the scaffold. Misassembled scaffolds in the central part of 7p (unaffected by the laser) show sharply uneven distribution of reads, with some areas showing values of ~30–40 hit/kb and others <1 hit/kb.

In our analysis of the v7.1 assembly, 91% of uniquely-mapping reads were to above-threshold (29 hits/kb) scaffolds, a lower percentage than when compared with the v4.1 assembly and map (see Table 2). Our analysis suggests that this is largely due to hits in a few defined regions that were misassembled in v7.1 chromosome-scale superscaffolds 1, 3 and 4, leading to overall below-threshold values for those entire chromosomes/scaffolds. Our analysis also identifies 97 above-threshold orphan scaffolds, contributing an additional 7.2 Mb of sequence to the 7p region.

**Table 2:** **Reads locating uniquely to *Xenopus tropicalis *v7.1 assembly**

**All hits (%)**	**Hits to v7.1 superscaffold 7 (%)**	**Hits to above-threshold orphan scaffolds (%)**	**Hits to under-threshold scaffolds (%)**
4,489,728	3,627,889	461,438	228,745
(100%)	(80.8%)	(10.26%)	(8.94%)

Threshold set to 29 hit/kb (hit/kb of scaffold 7).

Analysis of sequence from microdissected chromosome arms identified errors in both available *X. tropicalis* assemblies, with at least 15% of v4.1 scaffolds mapped to 7p scaffolds misassembled. In the v7.1 assembly, we located large regions of 7p sequence which were misassigned to superscaffolds 1, 3, and 4 (Additional file 6). Interestingly, v4.1 scaffolds_75, _266, and _270, identified as hybrids by our analysis, were divided in the v7.1 assembly. However, FISH analysis using probes corresponding to the *atp13a1* and *lacc1* genes from hit-absent regions of v4.1 scaffolds_75 and _266 revealed actual locations on chromosomes 3 and 4, respectively, rather than the positions given by the v7.1 assembly on chromosomes/superscaffolds 8 and 2. These results suggest that the v7.1 assembly has successfully identified bad sequence joins, but has not necessarily correctly repaired them in all cases. High throughput sequencing of microdissected chromosomes or chromosomal arms helps to identify such misassembled domains, as well as to assign orphan scaffolds to chromosomal regions. Microdissection and sequencing of particularly problematic areas, such as 7p and 2p, allows sequence domains to be assigned to a specific chromosome arm before extrapolating position from meiotic map or synteny data. Improving assembly of the short arm of chromosome 7 is critical for characterizing the sex-determination genes of *Xenopus tropicalis*, which is known to use a different system from the *DM-W* mechanism found in *X. laevis*[23].

Since our method could be combined with chromosome banding, it is likely to be particularly useful for *de novo* assembly of challenging genome projects, such as that of the allotetraploid laboratory model *Xenopus laevis* (*N =*18). In the absence of meiotic or physical map information, correct regional assembly and long-range contiguity would be enhanced by dissecting and sequencing specific chromosomes, all of which in *X. laevis* can be unambiguously distinguished by banding pattern [24].

## Conclusions

We have demonstrated feasibility of high-throughput sequencing from as little as 15 microdissected chromosome arms. This approach will be helpful for validating and completing problematic regions in the *X. tropicalis* genome, and can also be used in other species without sequenced genomes for describing gene content in selected chromosomes or providing long-range contiguity. Moreover, the technique is applicable to molecular analysis of isolated chromosomes from small numbers of cells, which is important for investigation of haplotypes or molecular rearrangements in clinical cytogenetics or oncology.

## Methods

### Chromosomal spreads for laser microdissection

*X. tropicalis* chromosome nomenclature followed [20]. Metaphase spreads were prepared from euploid primary cell cultures of dissected testes as described in [20] with minor changes. Cells were trypsinized and hypotonized in 38 mM KCl for 5 min. After fixation, cell suspensions were stored overnight at −20°C. For laser microdissection, cells were dropped on a polyethylene naphthalene membrane (P.A.L.M. GmbH, Bernried, Germany) attached to a thin glass slide, allowed to dry, and stained with 3% Giemsa in H_2_O for 10 min.

### Laser microdissection

Chromosomes were harvested as in [25]. Briefly, 15 copies of the p arm of chromosome 7 were microdissected and collected using a PALM MicroLaser system (P.A.L.M. GmbH, Bernried, Germany) coupled with an inverted microscope (Olympus) under an oil immersion objective (100× magnification). Chromosome arms were catapulted by a single laser pulse directly into the cap of a PCR tube containing 4 μL PCR oil. To ensure that the whole short arm of chromosome 7 was obtained, the laser cut was targeted to the q arm border of the centromere region.

### Whole genome amplification

An initial round of whole genome amplification was performed using the WGA4 *GenomePlex Single Cell Kit (Sigma-Aldrich)*. Dissected chromosome arms were digested with Proteinase K, followed by library preparation and amplification according to manufacturer’s instructions. Primary PCR products were cleaned up using a Qiaquick Gel Extraction Kit (QIAGEN) column. 20 ng of primary WGA4 product was then reamplified using the WGA3 system (*GenomePlex WGA Reamplification Kit, Sigma-Aldrich)* according to our original protocol for preparation of *X. tropicalis* painting probes [26]. The secondary PCR product was purified by ethanol precipitation, yielding approximately 20 μg of DNA fragments averaging ~500–600 bp in size.

### High throughput sequencing and library construction

In order to minimize resequencing WGA adaptors at ends of amplified fragments, libraries for high-throughput sequencing were constructed by *in vitro* transposition to simultaneously fragment the DNA and introduce sequencing primer/adaptors using the Nextera DNA sample prep kit (Illumina, Inc.) according to manufacturer’s instructions. 50 ng (measured by QuBit, Life Technology) of dissected chromosome amplification product DNA was used with Nextera Illumina-Compatible Enzyme Mix and low molecular weight buffer to generate libraries with fragment size of ~200 to 400 bp (including the 135 bp adapter sequence), and enriched by limited-cycle PCR. Library quality was determined by QuBit, Agilent Bioanalyser and QT-PCR using KAPA library quantification kit (KAPABiosystems, Boston, USA) before loading 6.5 pM on a lane of a GAII flow cell for sequencing 80 bp single reads.

### Data analysis

Reads were mapped to *Xenopus tropicalis* v4.1 and v7.1 assemblies (available on http://www.xenbase.org) [27] using Bowtie (http://bowtie-bio.sourceforge.net) [28,29]. Due to prevalence of repeats in the *X .tropicalis* genome, only unique hits with higher than 97.5% identity were selected, using Bowtie parameters m=1 and v=2. The sequences obtained were counted and analyzed using PERL scripts and Microsoft Excel. Repetitive genomic regions were obtained from the UCSC (browser http://genome.ucsc.edu/).

*FISH-TSA* (Fluorescence In Situ Hybridization with Tyramide Signal Amplification).

Metaphase spreads for FISH-TSA analysis were prepared from the same euploid testes cell cultures as for the laser microdissection procedure, using the FISH-TSA protocol described in [30].

## Competing interests

The authors declare that they have no competing interests.

## Authors’ contributions

VK, ES and LZ designed the project; ES, SK, HC, AS, and TT performed the experiments; ES, NO, VZ & MG analyzed the data; ES, VK and LZ wrote the paper. All authors read and approved the final manuscript.

## Supplementary Material

Additional file 1 **Reads mapping uniquely to v4.1 assembly.** Table showing all unique hits to v4.1 scaffolds, ordered by hit/kb ratio. Click here for file

Additional file 2 **Reads localizing uniquely to meiotically-mapped chromosome 7 scaffolds.** Chromosome 7 meiotic map with polymorphic markers and associated *Xenopus tropicalis* v4.1 scaffolds are shown with hits/kb from dissected chromosome 7p sequence. Read density decreases sharply near 72 cM, just beneath centromere at 69 cM +/− 1cM. Click here for file

Additional file 3 **Uniquely-mapped read distribution on physical map.** All genes previously mapped by FISH-TSA [8,10,20] are shown with associated chromosome/linkage group (chrom/LG), meiotic map position, cognate v4.1 scaffolds with hit/kb ratio, probe size (Ampl. Length), relative distance from centromere (RDC), and v7.1 position. All regions with high read density (17–80 hits/kb, marked in red) are located on 7p. FISH-TSA probes in all other regions (black) have <1 hit/kb. Click here for file

Additional file 4 **Reads mapping uniquely to v4.1 scaffolds.** The file includes all v4.1 scaffolds larger than 100 kb with hit/kb>17. Every panel contains one scaffold with its length scale, position of all genes, and visualization of reads. Click here for file

Additional file 5 **Reads mapping uniquely to v7.1 assembly.** Table showing all unique hits to v7.1 scaffolds. Chromosome-scale superscaffolds are shown top right, with orphan scaffolds on left ordered by decreasing unique reads/kb ratio. Click here for file

Additional file 6 **Read distribution on v7.1 scaffolds.** The file includes all scaffolds larger than 100 kb with unique reads/kb>29.23, including scaffold 35 and regions with high coverage in superscaffolds 1, 2, 3, 4 and 8. Scaffolds are ordered by ascending scaffold number. Superscaffold 7 is divided into several smaller parts. Each scaffold shows length scale and reads. Click here for file
