# Supplementary material for: Efficient high-throughput sequencing of a laser microdissected chromosome arm
Source: BMC Genomics. 2013 May 28;14:357. doi: 10.1186/1471-2164-14-357 (PMC3701504; doi:10.1186/1471-2164-14-357)
Supplement: Additional file 4 — Reads mapping uniquely to v4.1 scaffolds. The file includes all v4.1 scaffolds larger than 100 kb with hit/kb>17. Every panel contains one scaffold with its length scale, position of all genes, and visualization of reads. [file 1471-2164-14-357-S4.pdf]

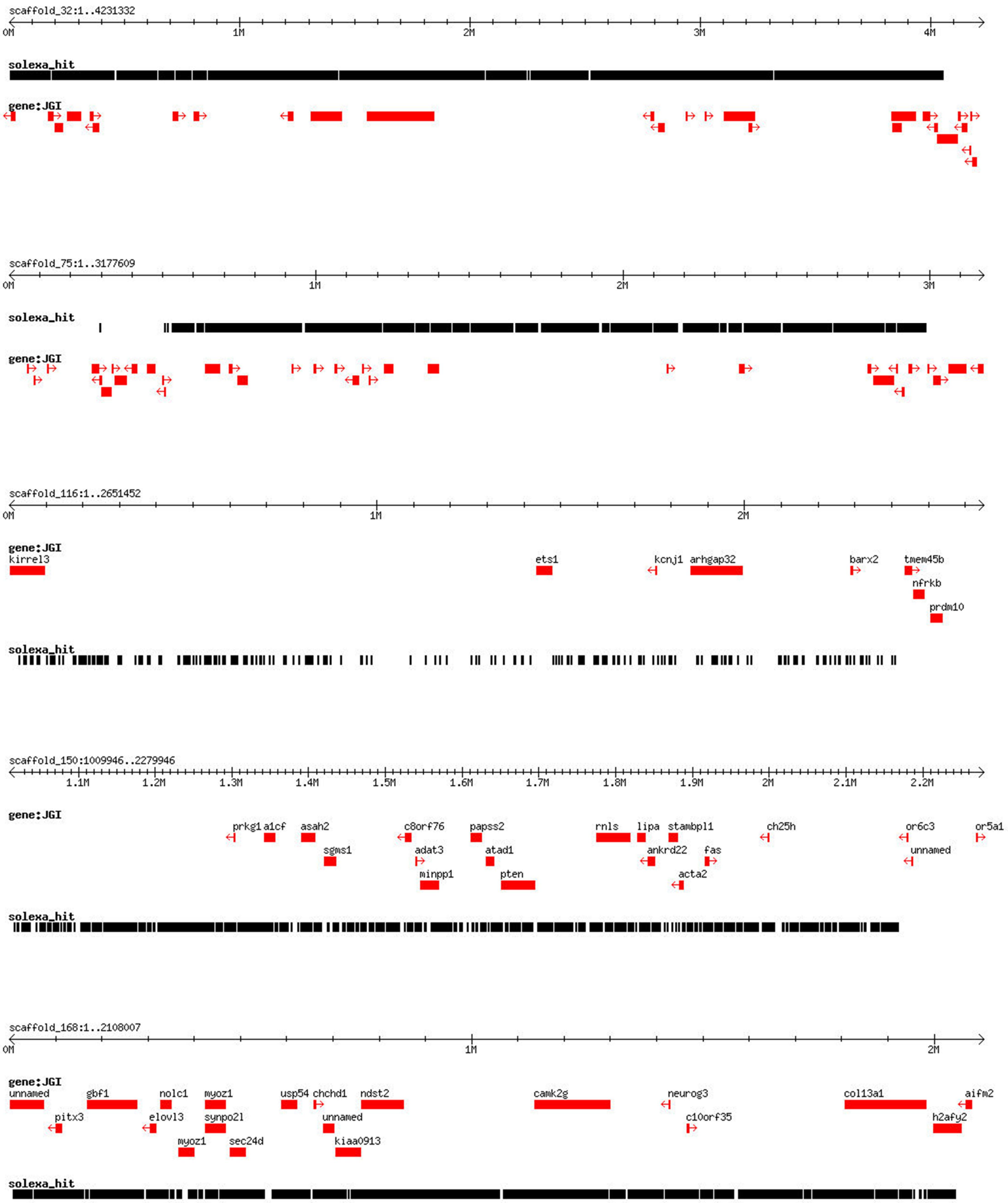
scaffold_32:1..4231332
0M
1M
2M
3M
4M
solexa_hit
gene:JGI
scaffold_75:1..3177609
0M
1M
2M
3M
solexa_hit
gene:JGI
scaffold_116:1..2651452
0M
1M
2M
gene:JGI
kirrel3
ets1
kcnj1
arhgap32
barx2
tmem45b
nfrkb
prdm10
solexa_hit
scaffold_150:1009946..2279946
1.1M
1.2M
1.3M
1.4M
1.5M
1.6M
1.7M
1.8M
1.9M
2M
2.1M
2.2M
gene:JGI
prkg1
a1cf
asah2
c8orf76
papss2
rnls
lipa
stambpl1
ch25h
or6c3
or5a1
sgms1
adat3
atad1
ankrd22
fas
unnamed
minpp1
pten
acta2
solexa_hit
scaffold_168:1..2108007
0M
1M
2M
gene:JGI
unnamed
gbf1
nolc1
myoz1
usp54
chchd1
ndst2
camk2g
neurog3
col13a1
aifm2
pitx3
elovl3
synpo2l
unnamed
c10orf35
h2afy2
myoz1
sec24d
kiaa0913
solexa_hit

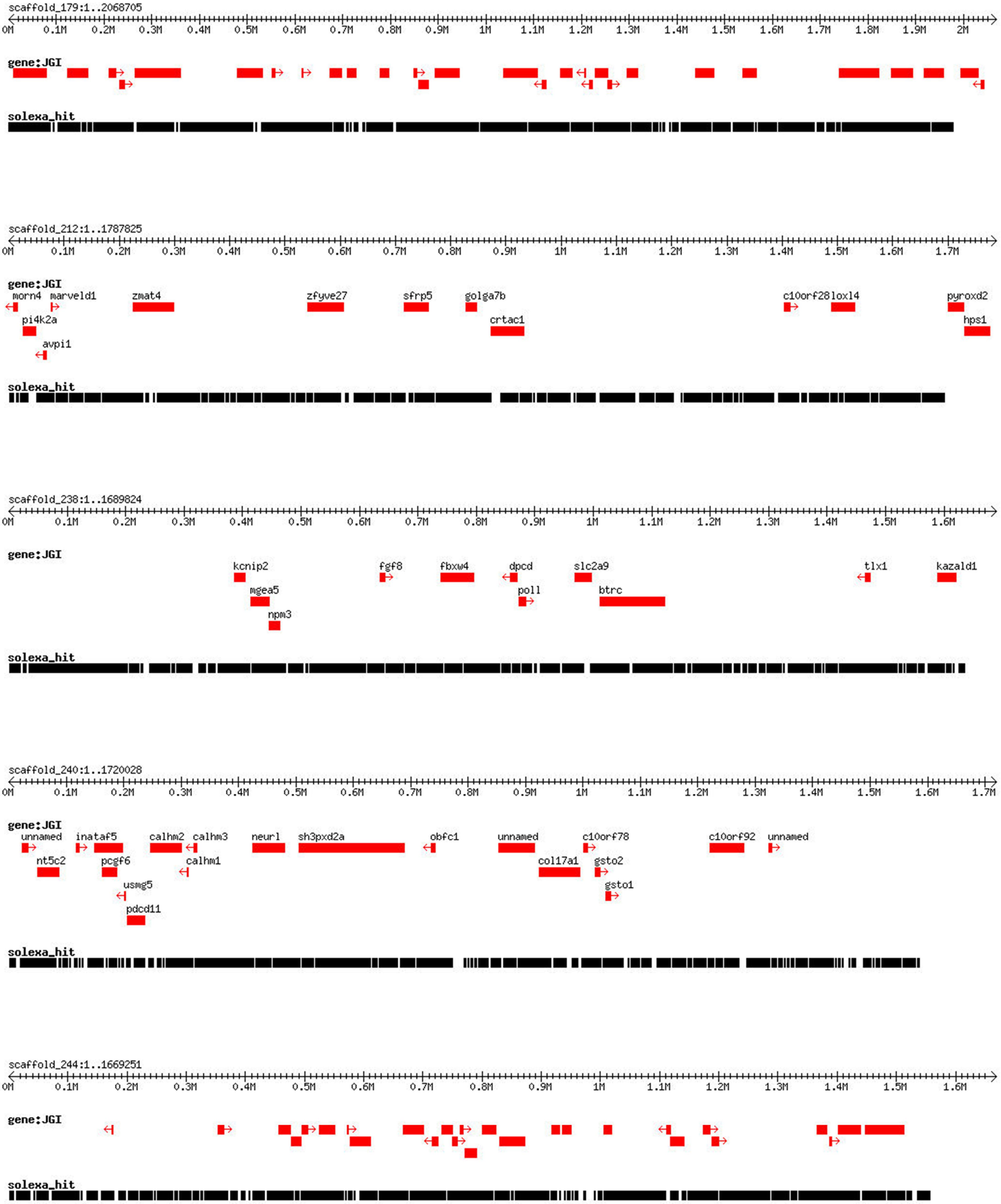
scaffold_179:1..2068705
0M 0.1M 0.2M 0.3M 0.4M 0.5M 0.6M 0.7M 0.8M 0.9M 1M 1.1M 1.2M 1.3M 1.4M 1.5M 1.6M 1.7M 1.8M 1.9M 2M
gene:JGI
solexa_hit
scaffold_212:1..1787825
0M 0.1M 0.2M 0.3M 0.4M 0.5M 0.6M 0.7M 0.8M 0.9M 1M 1.1M 1.2M 1.3M 1.4M 1.5M 1.6M 1.7M
gene:JGI
morn4
marveld1
zmat4
zfyve27
sfrp5
golga7b
c10orf28
lox14
pyroxd2
pi4k2a
crtac1
hps1
avpi1
solexa_hit
scaffold_238:1..1689824
0M 0.1M 0.2M 0.3M 0.4M 0.5M 0.6M 0.7M 0.8M 0.9M 1M 1.1M 1.2M 1.3M 1.4M 1.5M 1.6M
gene:JGI
kcnip2
fgf8
fbxw4
dpcd
slc2a9
tlx1
kazald1
mgea5
poll
btrc
npm3
solexa_hit
scaffold_240:1..1720028
0M 0.1M 0.2M 0.3M 0.4M 0.5M 0.6M 0.7M 0.8M 0.9M 1M 1.1M 1.2M 1.3M 1.4M 1.5M 1.6M 1.7M
gene:JGI
unnamed
inataf5
calhm2
calhm3
neurl
sh3pxd2a
obfc1
unnamed
c10orf78
c10orf92
unnamed
nt5c2
pcgf6
calhm1
col17a1
gsto2
usmg5
gsto1
pdcd11
solexa_hit
scaffold_244:1..1669251
0M 0.1M 0.2M 0.3M 0.4M 0.5M 0.6M 0.7M 0.8M 0.9M 1M 1.1M 1.2M 1.3M 1.4M 1.5M 1.6M
gene:JGI
solexa_hit

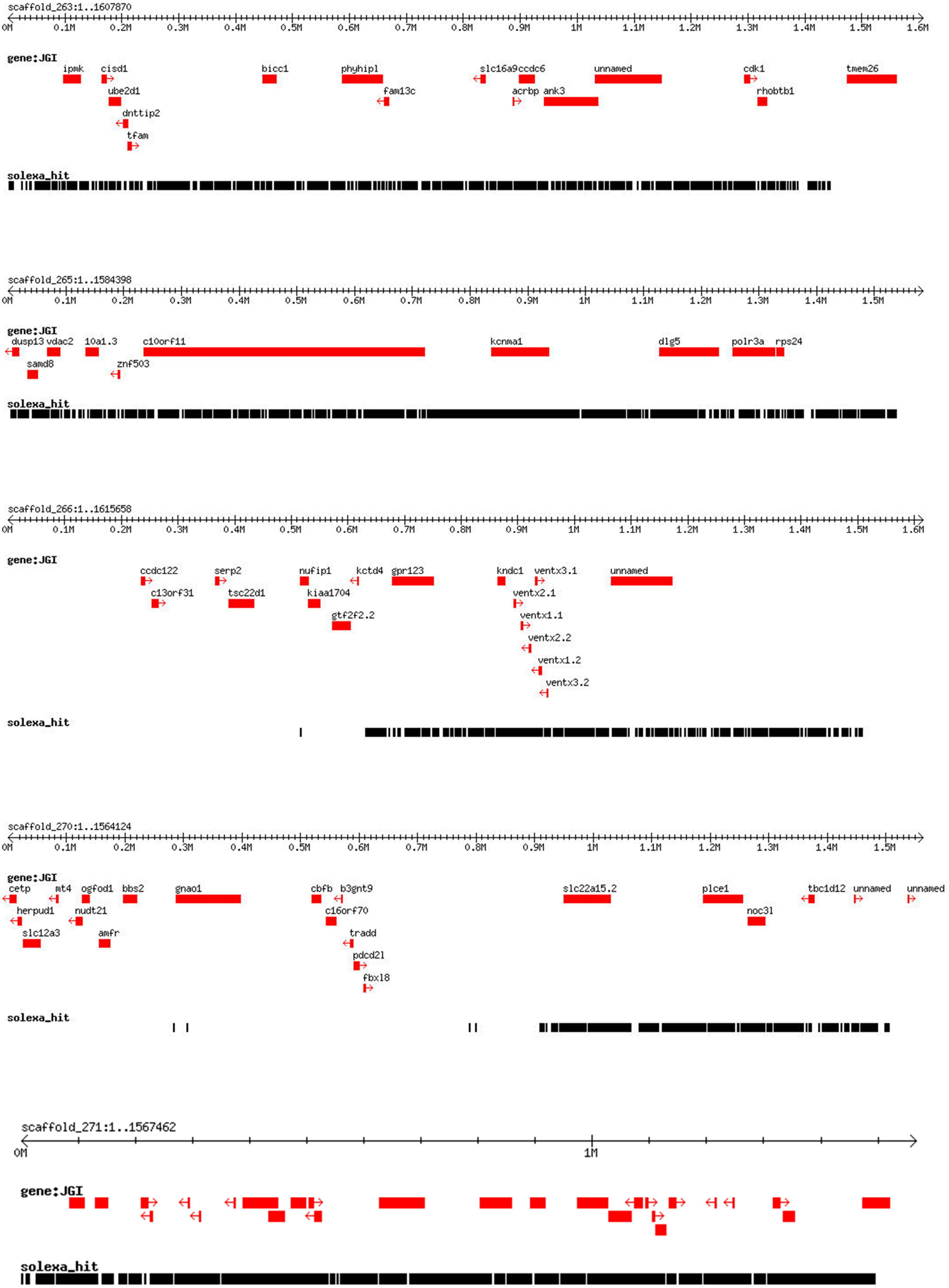

scaffold_263:1..1607870
0M 0.1M 0.2M 0.3M 0.4M 0.5M 0.6M 0.7M 0.8M 0.9M 1M 1.1M 1.2M 1.3M 1.4M 1.5M 1.6M
gene:JGI
ipmk cisd1 bicc1 phyhipl slc16a9ccdc6 unnamed cdk1 tmem26
ube2d1 fam13c acrbp ank3 rhobtb1
dnttip2
tfam
solexa_hit
scaffold_265:1..1584398
0M 0.1M 0.2M 0.3M 0.4M 0.5M 0.6M 0.7M 0.8M 0.9M 1M 1.1M 1.2M 1.3M 1.4M 1.5M
gene:JGI
dusp13 vdac2 10a1.3 c10orf11 kcnma1 dlg5 polr3a rps24
samd8 znf503
solexa_hit
scaffold_266:1..1615658
0M 0.1M 0.2M 0.3M 0.4M 0.5M 0.6M 0.7M 0.8M 0.9M 1M 1.1M 1.2M 1.3M 1.4M 1.5M 1.6M
gene:JGI
ccdc122 serp2 nufip1 kctd4 gpr123 kndc1 ventx3.1 unnamed
c13orf31 tsc22d1 kiaa1704 ventx2.1
gtf2f2.2 ventx1.1
ventx2.2
ventx1.2
ventx3.2
solexa_hit
scaffold_270:1..1564124
0M 0.1M 0.2M 0.3M 0.4M 0.5M 0.6M 0.7M 0.8M 0.9M 1M 1.1M 1.2M 1.3M 1.4M 1.5M
gene:JGI
cetp mt4 ogfod1 bbs2 gnao1 cbfb b3gnt9 slc22a15.2 plce1 tbc1d12 unnamed unnamed
herpud1 nudt21 c16orf70 noc3l
slc12a3 amfr tradd
pdcd2l
fbxl8
solexa_hit
scaffold_271:1..1567462
0M 1M
gene:JGI
solexa_hit

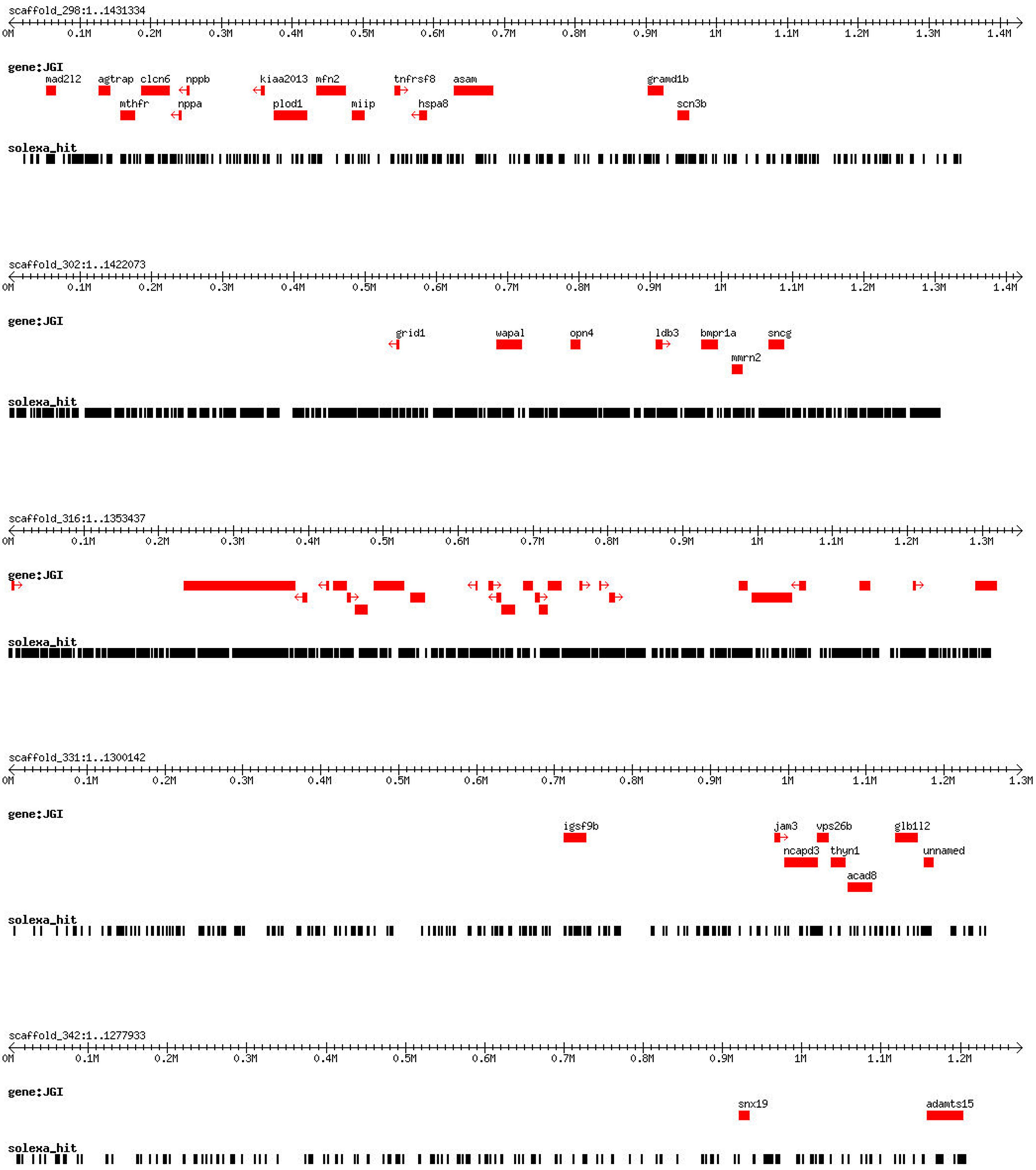
scaffold_298:1..1431334
0M 0.1M 0.2M 0.3M 0.4M 0.5M 0.6M 0.7M 0.8M 0.9M 1M 1.1M 1.2M 1.3M 1.4M
gene:JGI
mad2l2 agtrap clcn6 nppb kiaa2013 mfn2 tnfrsf8 asam gramd1b
mthfr nppa plod1 miip hspa8 scn3b
solexa_hit
scaffold_302:1..1422073
0M 0.1M 0.2M 0.3M 0.4M 0.5M 0.6M 0.7M 0.8M 0.9M 1M 1.1M 1.2M 1.3M 1.4M
gene:JGI
grid1 wapal opn4 ldb3 bmpr1a sncg
mmrn2
solexa_hit
scaffold_316:1..1353437
0M 0.1M 0.2M 0.3M 0.4M 0.5M 0.6M 0.7M 0.8M 0.9M 1M 1.1M 1.2M 1.3M
gene:JGI
solexa_hit
scaffold_331:1..1300142
0M 0.1M 0.2M 0.3M 0.4M 0.5M 0.6M 0.7M 0.8M 0.9M 1M 1.1M 1.2M 1.3M
gene:JGI
igsf9b jam3 vps26b glb1l2
ncapd3 thyn1 unnamed
acad8
solexa_hit
scaffold_342:1..1277933
0M 0.1M 0.2M 0.3M 0.4M 0.5M 0.6M 0.7M 0.8M 0.9M 1M 1.1M 1.2M
gene:JGI
snx19 adamts15
solexa_hit

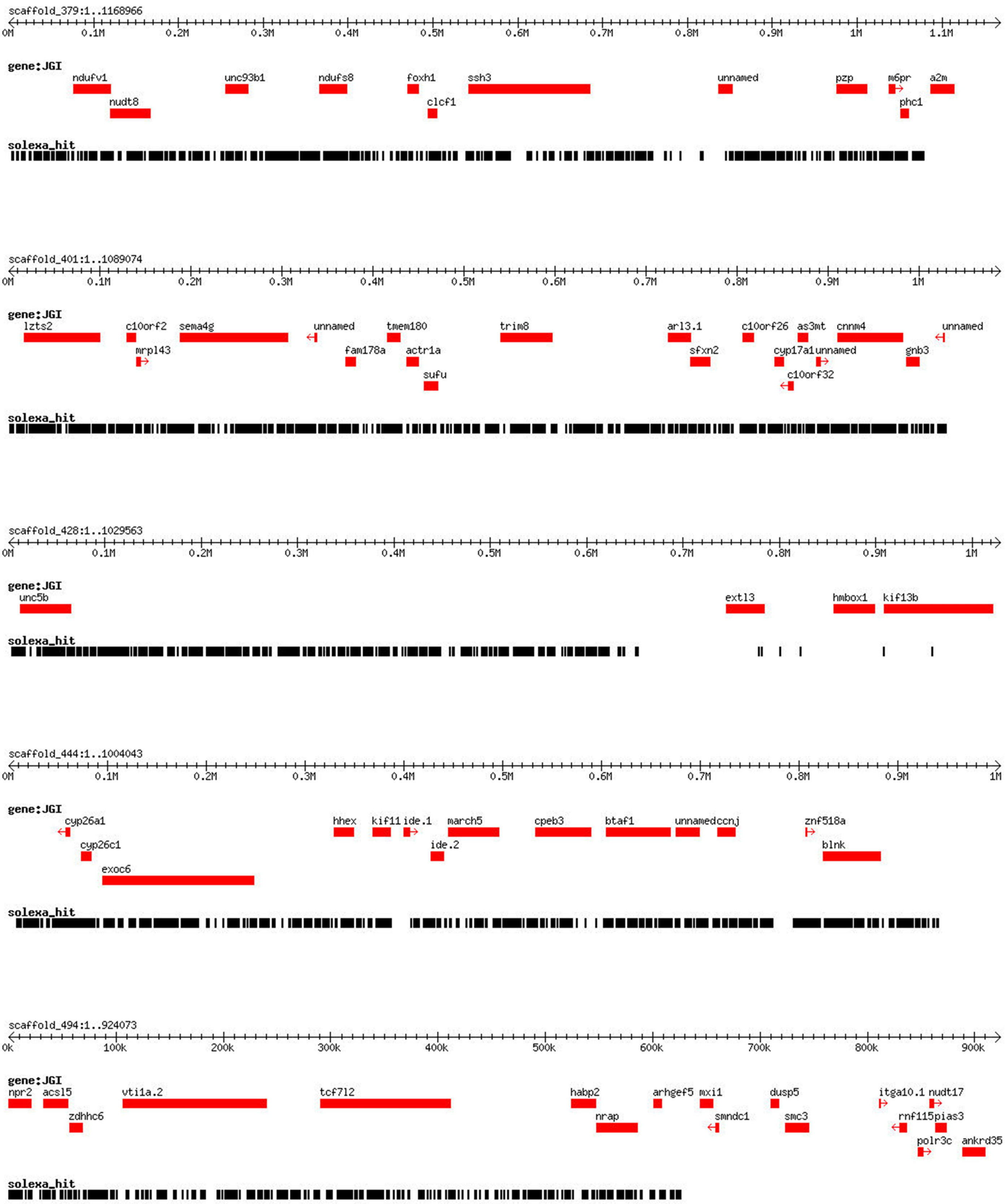
scaffold_379:1..1168966
0M 0.1M 0.2M 0.3M 0.4M 0.5M 0.6M 0.7M 0.8M 0.9M 1M 1.1M
gene:JGI
ndufv1 unc93b1 ndufs8 foxh1 ssh3 unnamed pzp m6pr a2m
nudt8 clcf1 phc1
solexa_hit
scaffold_401:1..1089074
0M 0.1M 0.2M 0.3M 0.4M 0.5M 0.6M 0.7M 0.8M 0.9M 1M
gene:JGI
lzts2 c10orf2 sema4g unnamed tmem180 trim8 arl3.1 c10orf26 as3mt cnnm4 unnamed
mrpl43 fam178a actr1a sfxn2 cyp17a1 unnamed gnb3
sufu c10orf32
solexa_hit
scaffold_428:1..1029563
0M 0.1M 0.2M 0.3M 0.4M 0.5M 0.6M 0.7M 0.8M 0.9M 1M
gene:JGI
unc5b extl3 hmbox1 kif13b
solexa_hit
scaffold_444:1..1004043
0M 0.1M 0.2M 0.3M 0.4M 0.5M 0.6M 0.7M 0.8M 0.9M 1M
gene:JGI
cyp26a1 hhex kif11 ide.1 march5 cpeb3 btaf1 unnamed ccnj znf518a
cyp26c1 ide.2 blnk
exoc6
solexa_hit
scaffold_494:1..924073
0k 100k 200k 300k 400k 500k 600k 700k 800k 900k
gene:JGI
npr2 acsl5 vti1a.2 tcf7l2 habp2 arhgef5 mxi1 dusp5 itga10.1 nudt17
zdhhc6 nrap smndc1 smc3 rnf115 pias3
polr3c ankrd35
solexa_hit

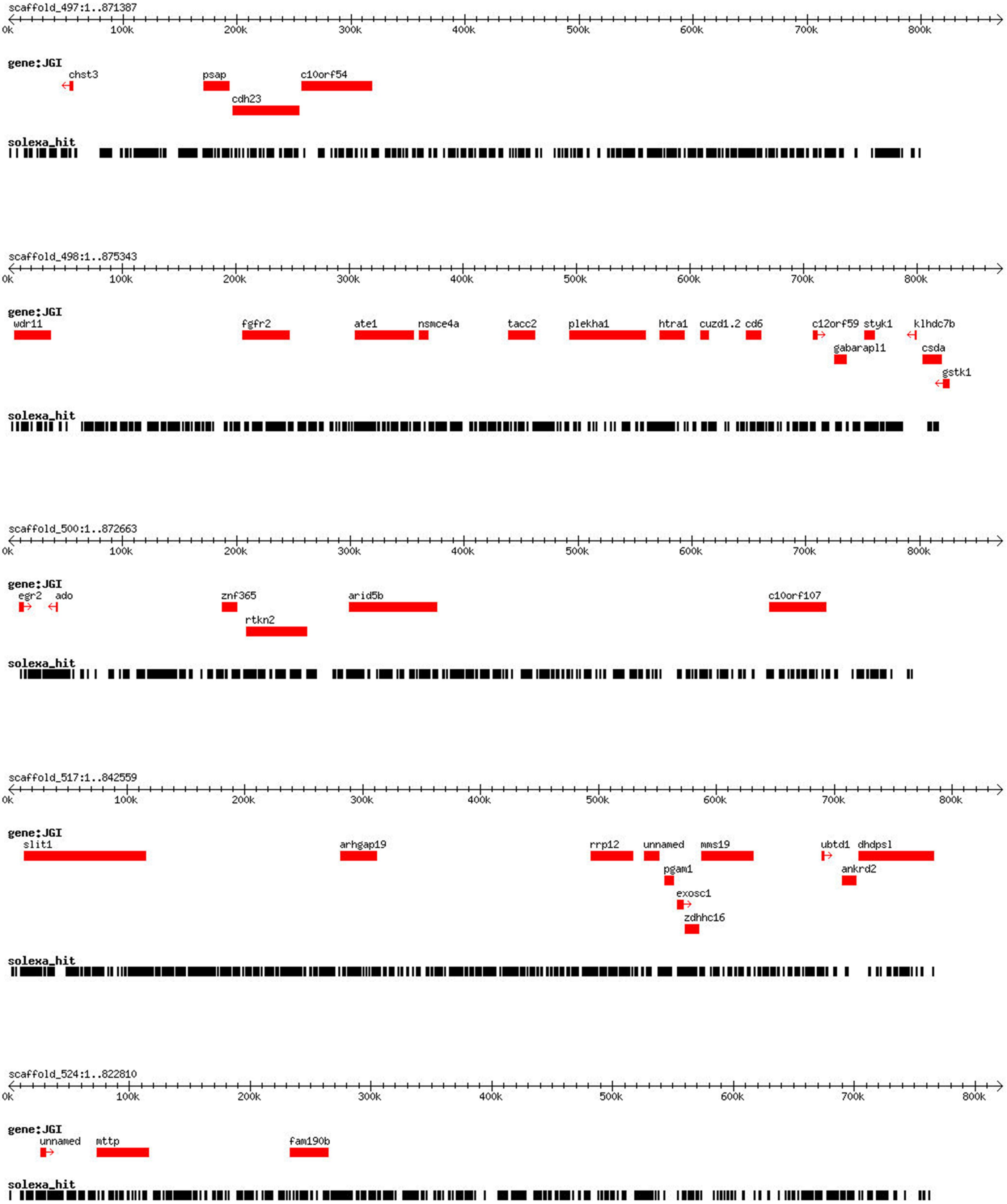
scaffold_497:1..871387
0k 100k 200k 300k 400k 500k 600k 700k 800k
gene:JGI
chst3
psap
c10orf54
cdh23
solexa_hit
scaffold_498:1..875343
0k 100k 200k 300k 400k 500k 600k 700k 800k
gene:JGI
wdr11
fgfr2
ate1
nsmce4a
tacc2
plekha1
htra1
cuzd1.2
cd6
c12orf59
styk1
klhdc7b
gabarapl1
csda
gstk1
solexa_hit
scaffold_500:1..872663
0k 100k 200k 300k 400k 500k 600k 700k 800k
gene:JGI
egr2
ado
znf365
arid5b
c10orf107
rtkn2
solexa_hit
scaffold_517:1..842559
0k 100k 200k 300k 400k 500k 600k 700k 800k
gene:JGI
slit1
arhgap19
rrp12
unnamed
mms19
ubtd1
dhdpsl
pgam1
ankrd2
exosc1
zdhhc16
solexa_hit
scaffold_524:1..822810
0k 100k 200k 300k 400k 500k 600k 700k 800k
gene:JGI
unnamed
mttp
fam190b
solexa_hit

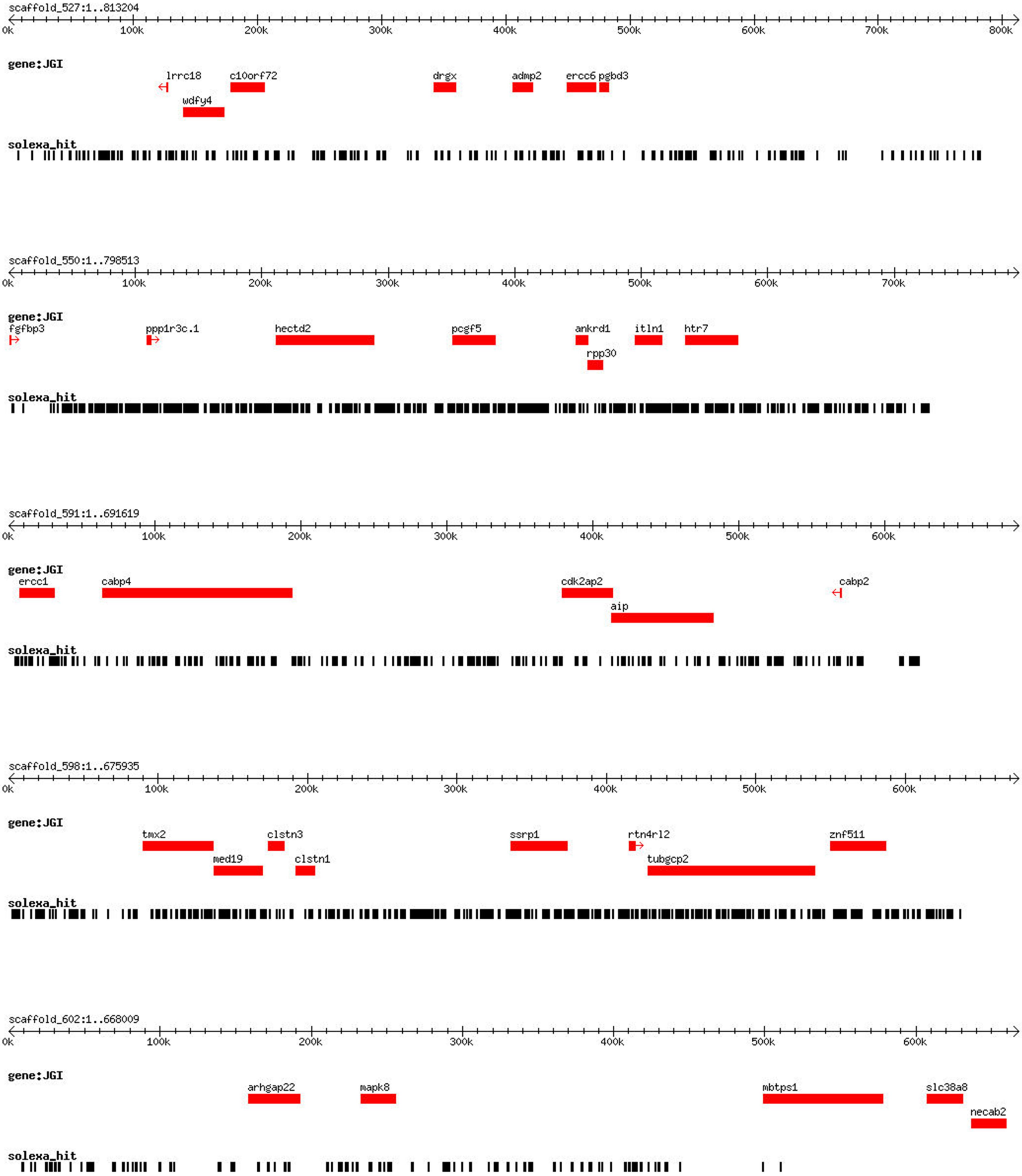
scaffold_527:1..813204
0k 100k 200k 300k 400k 500k 600k 700k 800k
gene:JGI
lrrc18
c10orf72
drgx
admp2
ercc6
pgbd3
wdfy4
solexa_hit
scaffold_550:1..798513
0k 100k 200k 300k 400k 500k 600k 700k
gene:JGI
fgfbp3
ppp1r3c.1
hectd2
pcgf5
ankrd1
itln1
htr7
rpp30
solexa_hit
scaffold_591:1..691619
0k 100k 200k 300k 400k 500k 600k
gene:JGI
ercc1
cabp4
cdk2ap2
cabp2
aip
solexa_hit
scaffold_598:1..675935
0k 100k 200k 300k 400k 500k 600k
gene:JGI
tmx2
clstn3
ssrp1
rtn4rl2
znf511
med19
clstn1
tubgcp2
solexa_hit
scaffold_602:1..668009
0k 100k 200k 300k 400k 500k 600k
gene:JGI
arhgap22
mapk8
mbtps1
slc38a8
necab2
solexa_hit

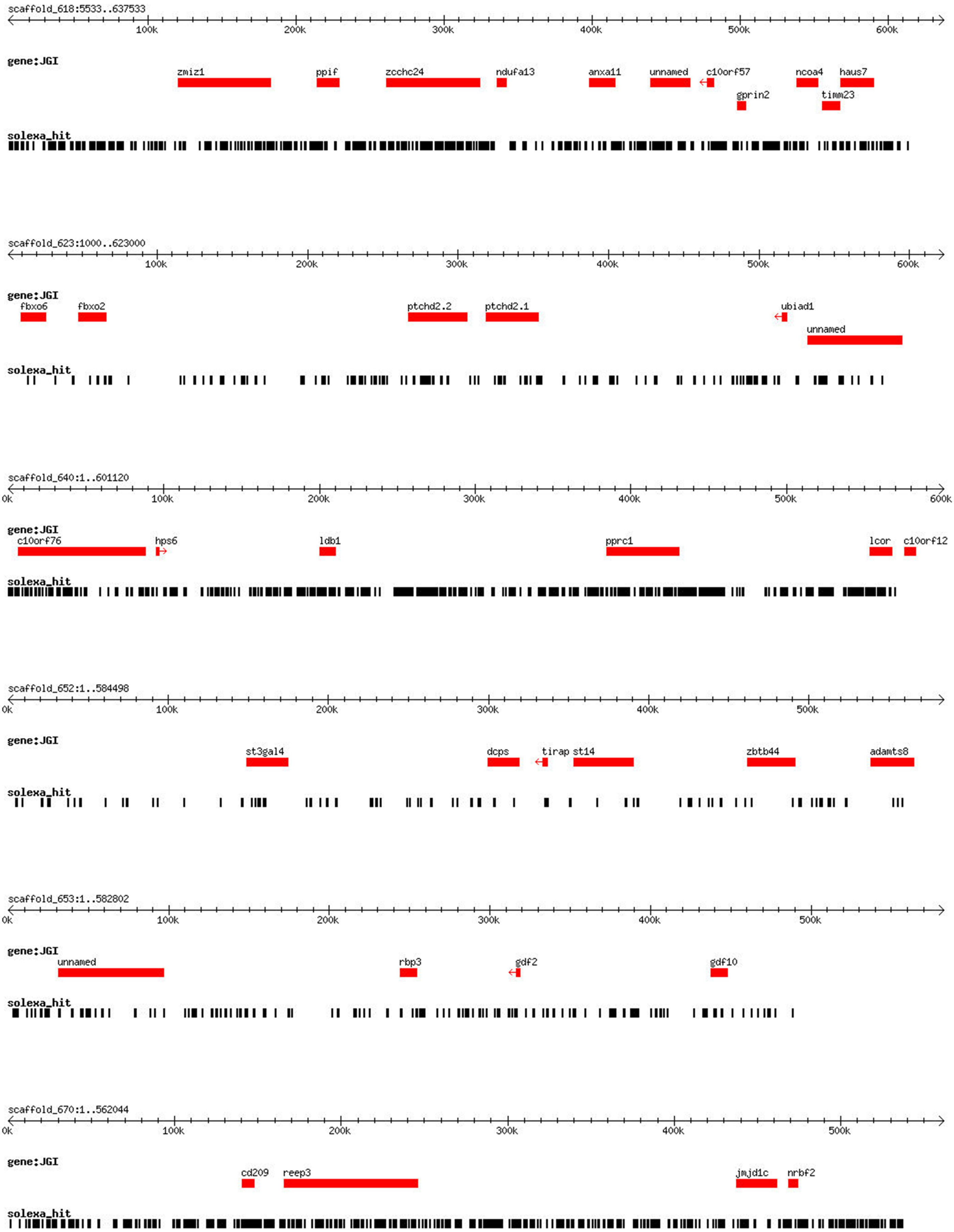
scaffold_618:5533..637533
100k
200k
300k
400k
500k
600k
gene:JGI
zmiz1
ppif
zcchc24
ndufa13
anxa11
unnamed
c10orf57
ncoa4
haus7
gprin2
timm23
solexa_hit
scaffold_623:1000..623000
100k
200k
300k
400k
500k
600k
gene:JGI
fbxo6
fbxo2
ptchd2.2
ptchd2.1
ubiad1
unnamed
solexa_hit
scaffold_640:1..601120
0k
100k
200k
300k
400k
500k
600k
gene:JGI
c10orf76
hps6
ldb1
pprc1
lcor
c10orf12
solexa_hit
scaffold_652:1..584498
0k
100k
200k
300k
400k
500k
gene:JGI
st3gal4
dcps
tirap
st14
zbtb44
adamts8
solexa_hit
scaffold_653:1..582802
0k
100k
200k
300k
400k
500k
gene:JGI
unnamed
rbp3
gdf2
gdf10
solexa_hit
scaffold_670:1..562044
0k
100k
200k
300k
400k
500k
gene:JGI
cd209
reep3
jmjd1c
nrbf2
solexa_hit

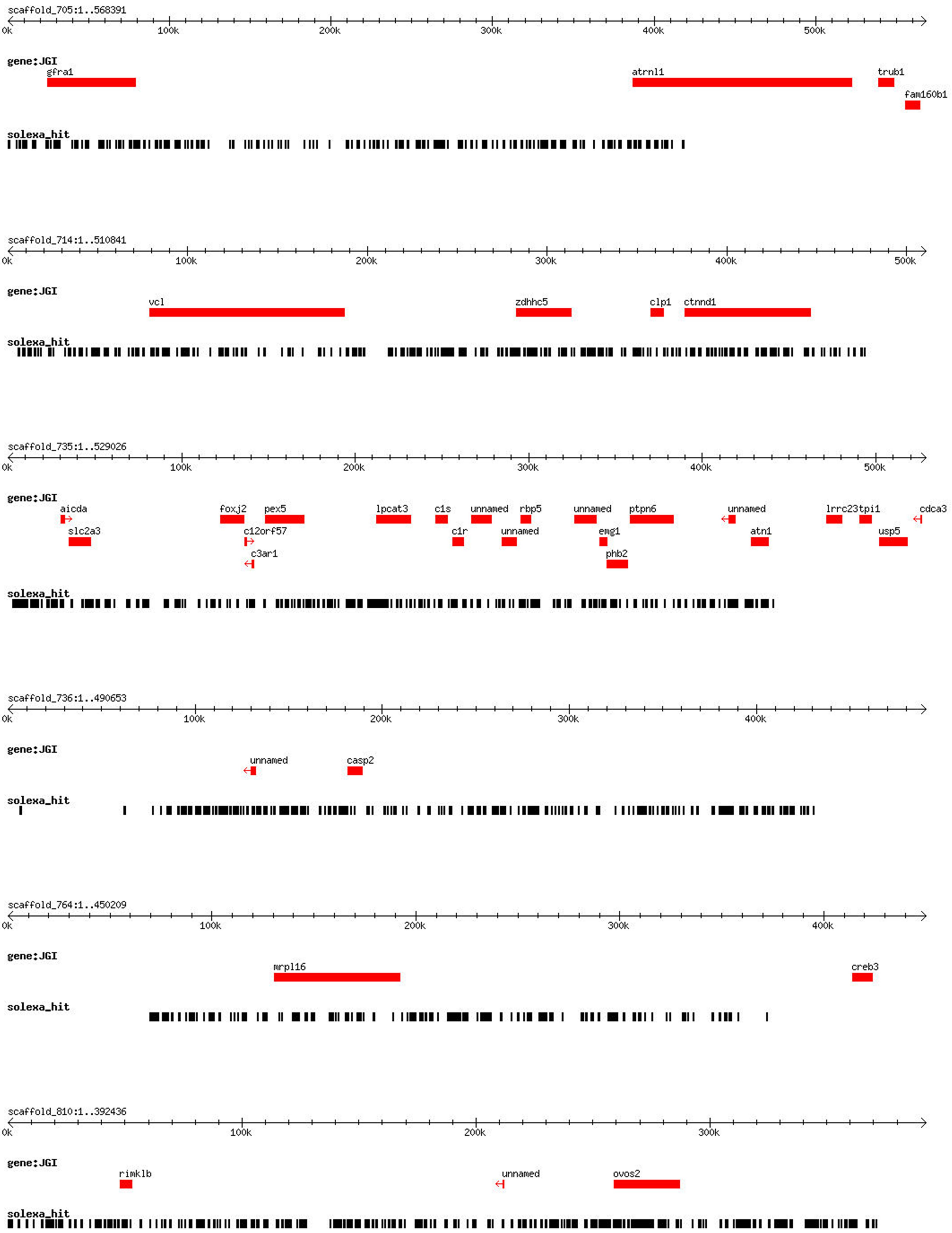

scaffold_705:1..568391
0k 100k 200k 300k 400k 500k
gene:JGI
gfra1
atrnl1
trub1
fam160b1
solexa_hit
scaffold_714:1..510841
0k 100k 200k 300k 400k 500k
gene:JGI
vcl
zdhhc5
clp1
ctnnd1
solexa_hit
scaffold_735:1..529026
0k 100k 200k 300k 400k 500k
gene:JGI
aicda
slc2a3
foxj2
pex5
c12orf57
c3ar1
lpcat3
c1s
c1r
unnamed
rbp5
unnamed
unnamed
emg1
phb2
ptpn6
unnamed
atn1
lrrc23
tpi1
usp5
cdca3
solexa_hit
scaffold_736:1..490653
0k 100k 200k 300k 400k
gene:JGI
unnamed
casp2
solexa_hit
scaffold_764:1..450209
0k 100k 200k 300k 400k
gene:JGI
mrpl16
creb3
solexa_hit
scaffold_810:1..392436
0k 100k 200k 300k
gene:JGI
rimklb
unnamed
ovos2
solexa_hit

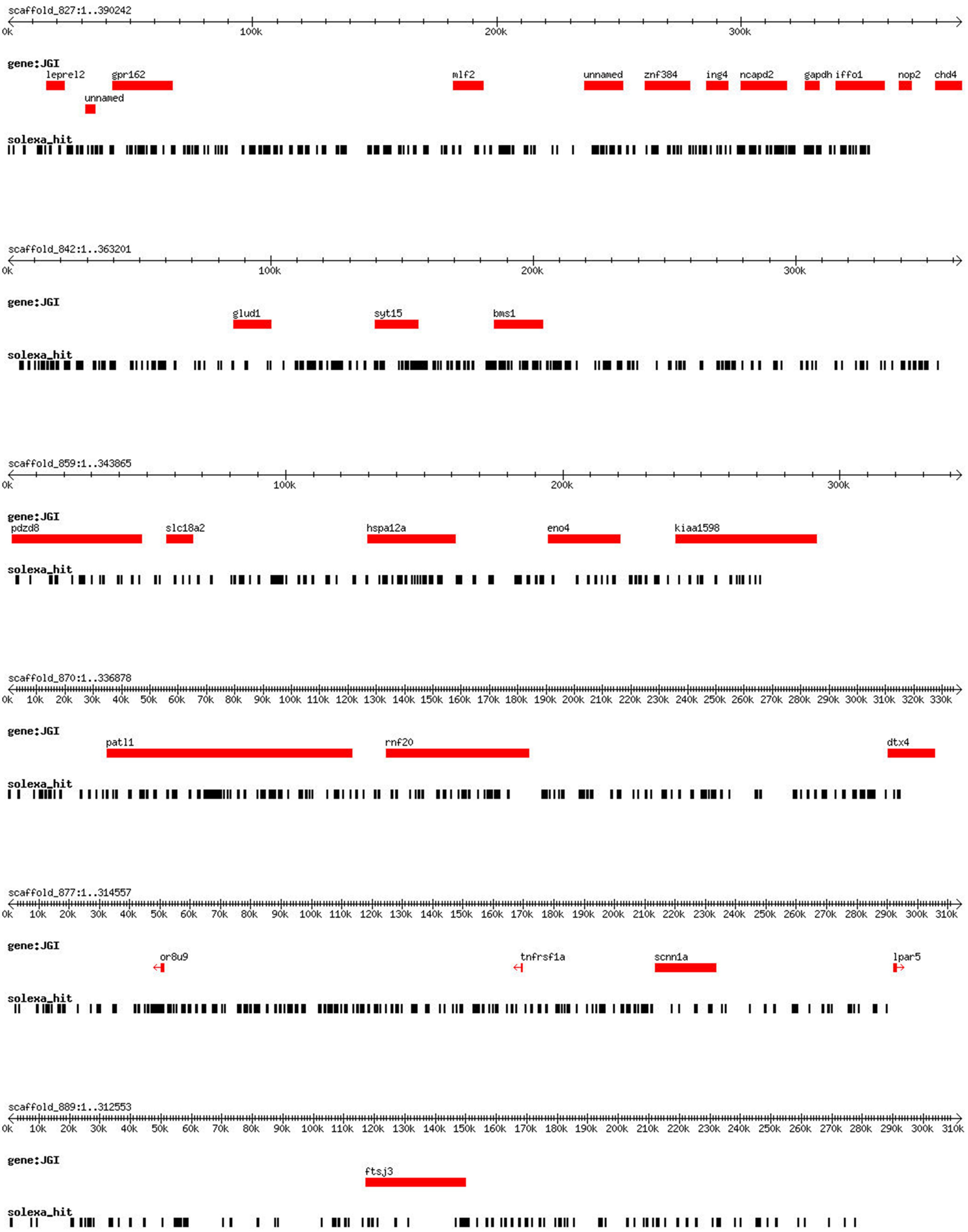
scaffold_827:1..390242
0k
100k
200k
300k
gene:JGI
leprel2
gpr162
unnamed
mlf2
unnamed
znf384
ing4
ncapd2
gapdh
iffo1
nop2
chd4
solexa_hit
scaffold_842:1..363201
0k
100k
200k
300k
gene:JGI
glud1
syt15
bms1
solexa_hit
scaffold_859:1..343865
0k
100k
200k
300k
gene:JGI
pdzd8
slc18a2
hspa12a
eno4
kiaa1598
solexa_hit
scaffold_870:1..336878
0k 10k 20k 30k 40k 50k 60k 70k 80k 90k 100k 110k 120k 130k 140k 150k 160k 170k 180k 190k 200k 210k 220k 230k 240k 250k 260k 270k 280k 290k 300k 310k 320k 330k
gene:JGI
patl1
rnf20
dtx4
solexa_hit
scaffold_877:1..314557
0k 10k 20k 30k 40k 50k 60k 70k 80k 90k 100k 110k 120k 130k 140k 150k 160k 170k 180k 190k 200k 210k 220k 230k 240k 250k 260k 270k 280k 290k 300k 310k
gene:JGI
or8u9
tnfrsf1a
scnn1a
lpar5
solexa_hit
scaffold_889:1..312553
0k 10k 20k 30k 40k 50k 60k 70k 80k 90k 100k 110k 120k 130k 140k 150k 160k 170k 180k 190k 200k 210k 220k 230k 240k 250k 260k 270k 280k 290k 300k 310k
gene:JGI
ftsj3
solexa_hit

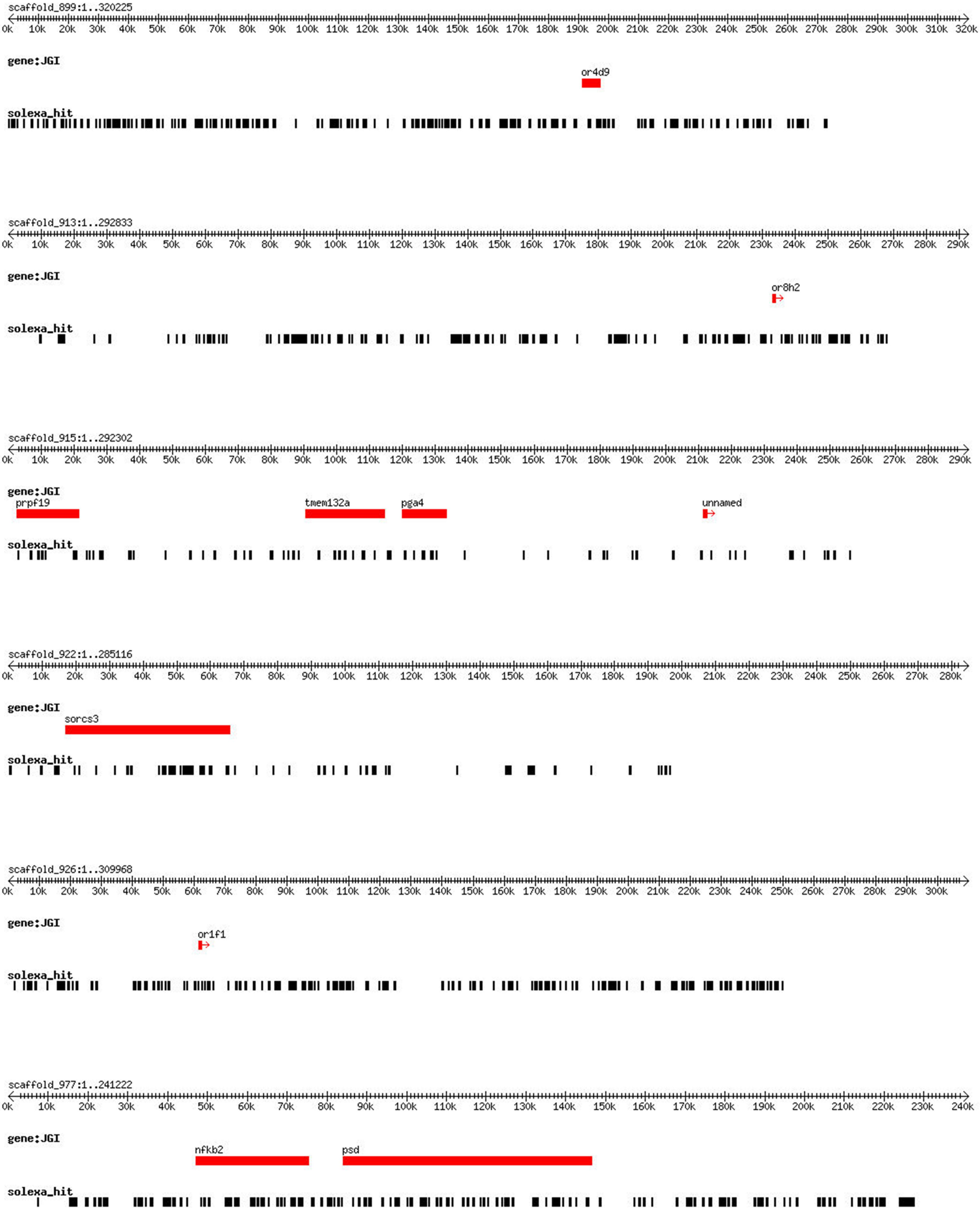

scaffold_899:1..320225
gene:JGI
or4d9
solexa_hit
scaffold_913:1..292833
gene:JGI
or8h2
solexa_hit
scaffold_915:1..292302
gene:JGI
prpf19
tmem132a
pga4
unnamed
solexa_hit
scaffold_922:1..285116
gene:JGI
sorcs3
solexa_hit
scaffold_926:1..309968
gene:JGI
or1f1
solexa_hit
scaffold_977:1..241222
gene:JGI
nfkb2
psd
solexa_hit

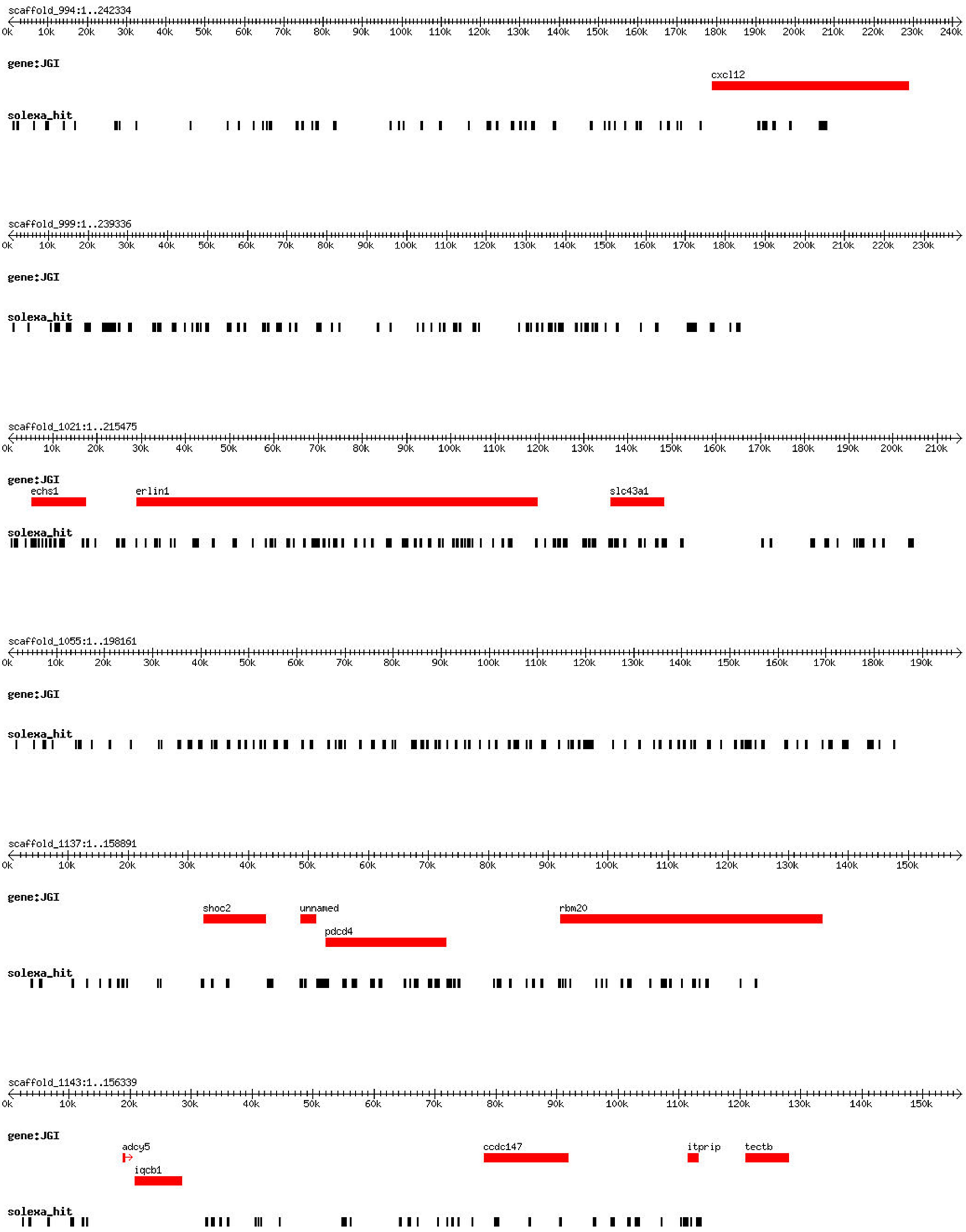
scaffold_994:1..242334
gene:JGI
cxcl12
solexa_hit
scaffold_999:1..239336
gene:JGI
solexa_hit
scaffold_1021:1..215475
gene:JGI
echs1
erlin1
slc43a1
solexa_hit
scaffold_1055:1..198161
gene:JGI
solexa_hit
scaffold_1137:1..158891
gene:JGI
shoc2
unnamed
pdcd4
rbm20
solexa_hit
scaffold_1143:1..156339
gene:JGI
adcy5
iqcb1
ccdc147
itprip
tectb
solexa_hit

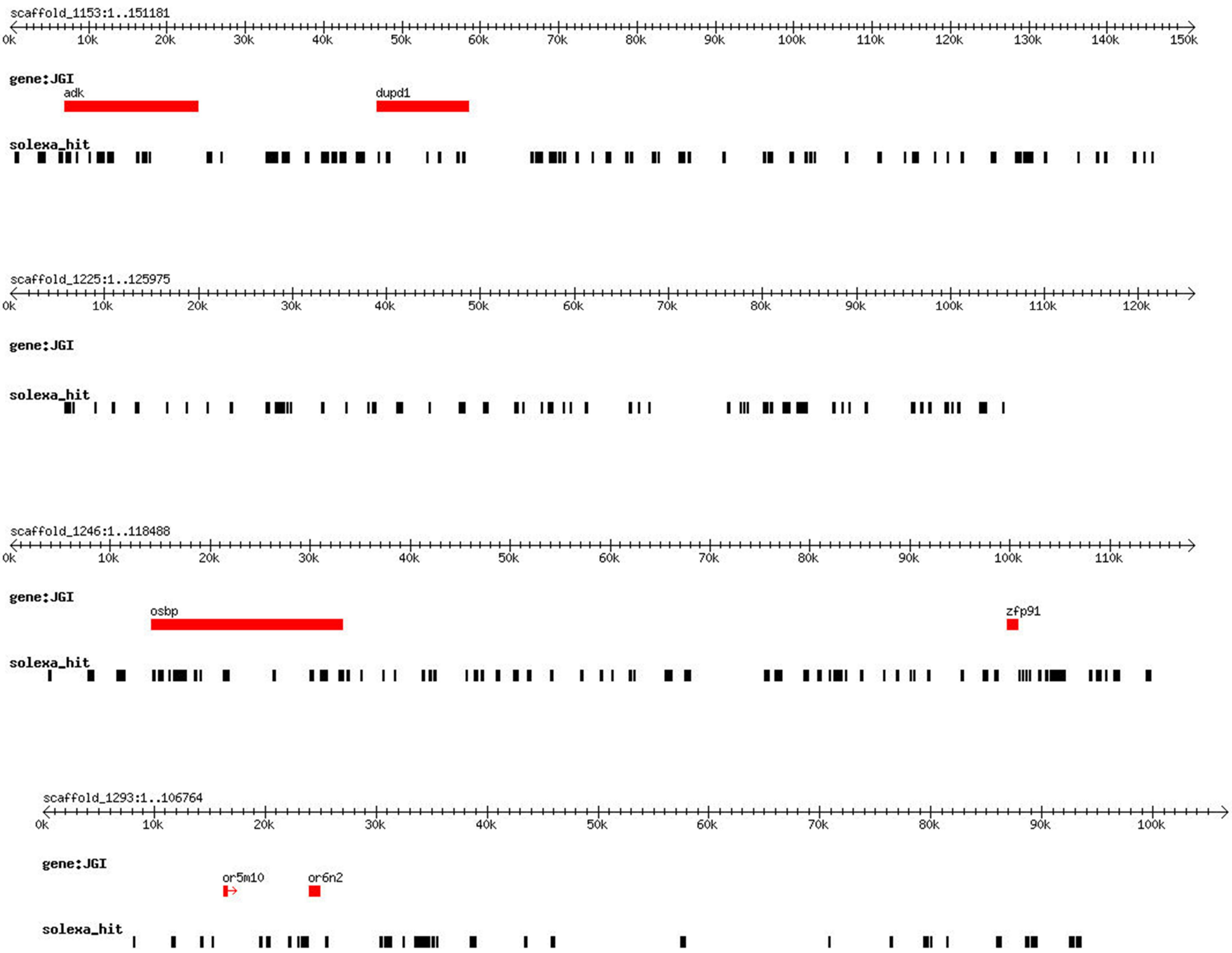

scaffold_1153:1..151181
0k 10k 20k 30k 40k 50k 60k 70k 80k 90k 100k 110k 120k 130k 140k 150k
gene:JGI
adk
dupd1
solexa_hit
scaffold_1225:1..125975
0k 10k 20k 30k 40k 50k 60k 70k 80k 90k 100k 110k 120k
gene:JGI
solexa_hit
scaffold_1246:1..118488
0k 10k 20k 30k 40k 50k 60k 70k 80k 90k 100k 110k
gene:JGI
osbp
zfp91
solexa_hit
scaffold_1293:1..106764
0k 10k 20k 30k 40k 50k 60k 70k 80k 90k 100k
gene:JGI
or5m10
or6n2
solexa_hit
