# Supplementary figures and images for: Efficient high-throughput sequencing of a laser microdissected chromosome arm
Source: BMC Genomics. 2013 May 28;14:357. doi: 10.1186/1471-2164-14-357 (PMC3701504; doi:10.1186/1471-2164-14-357)

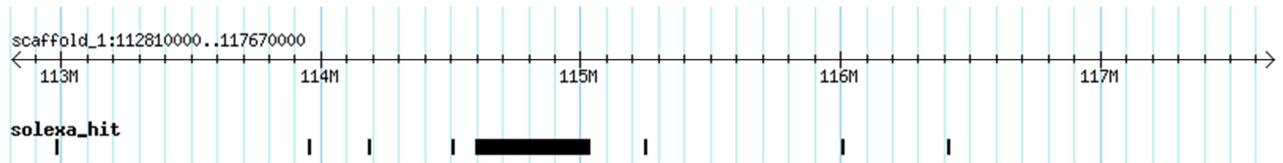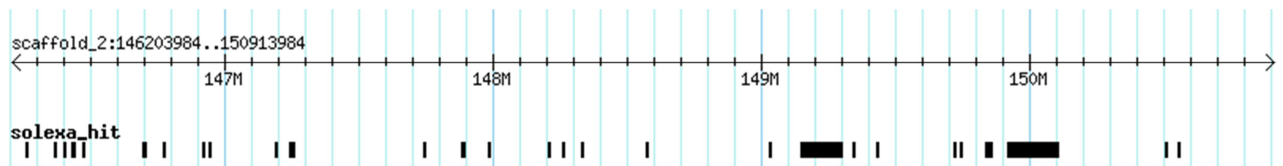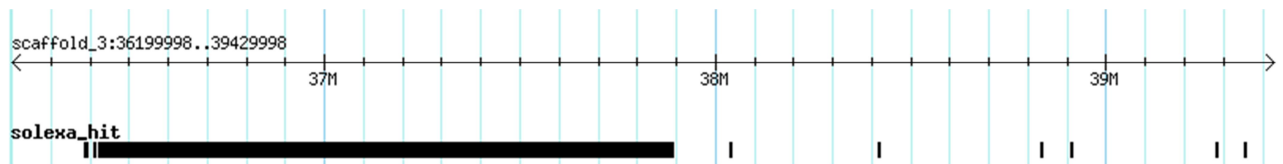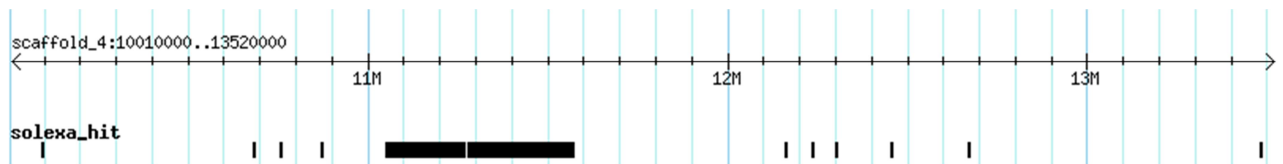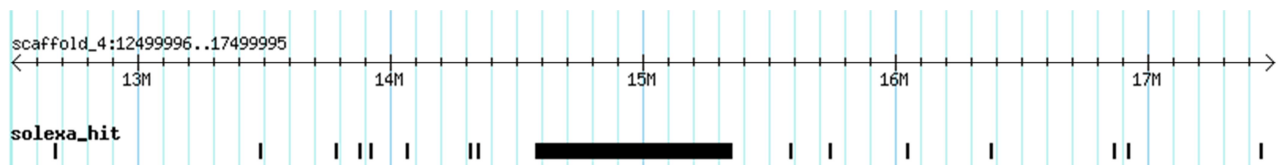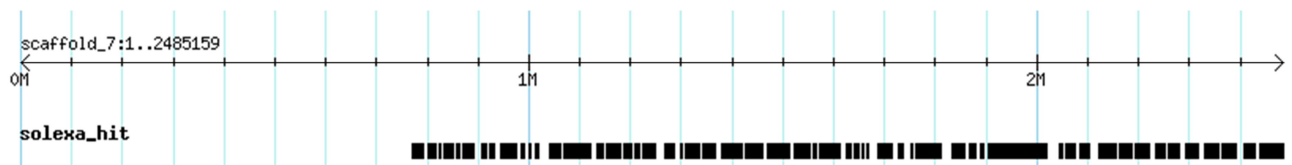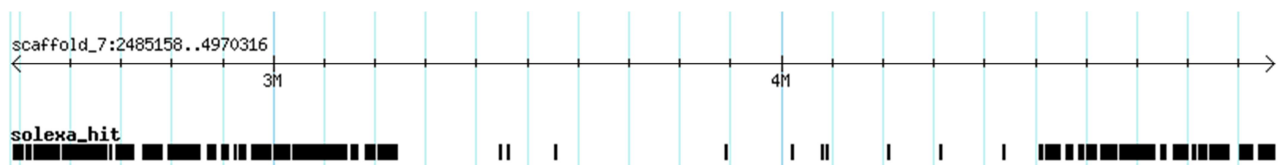

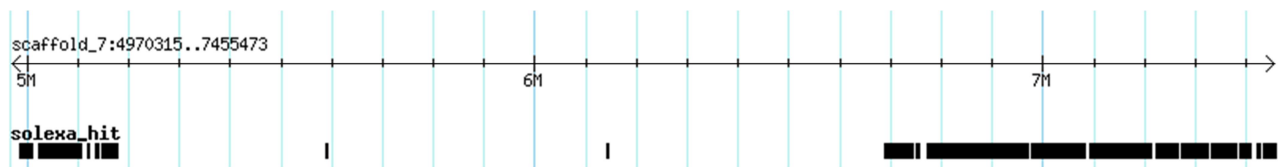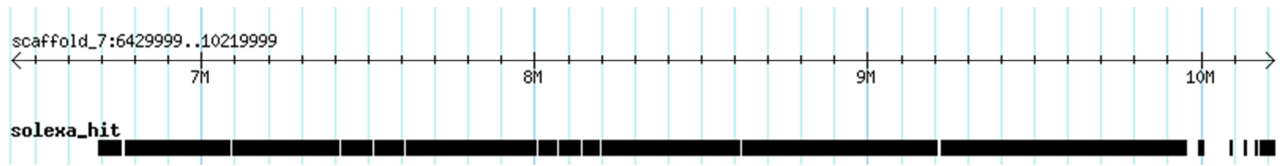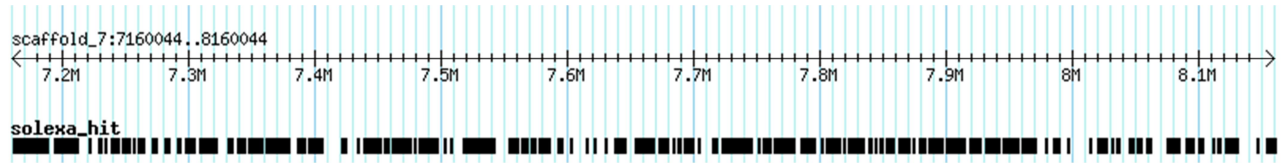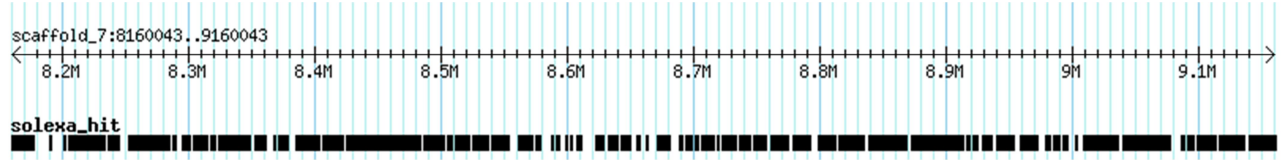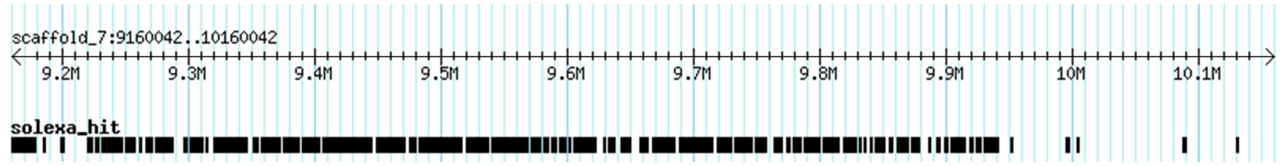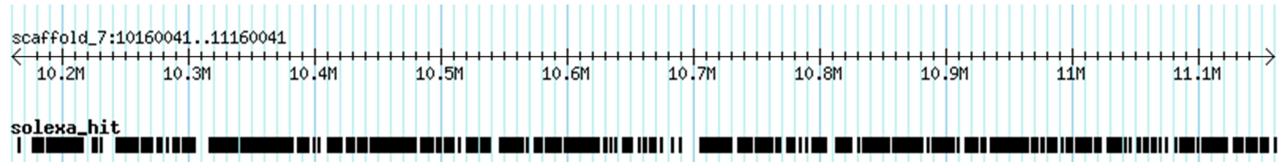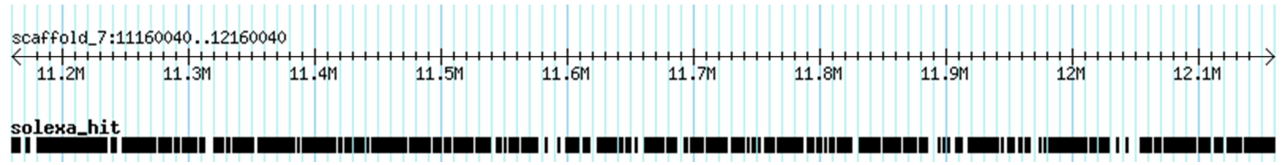

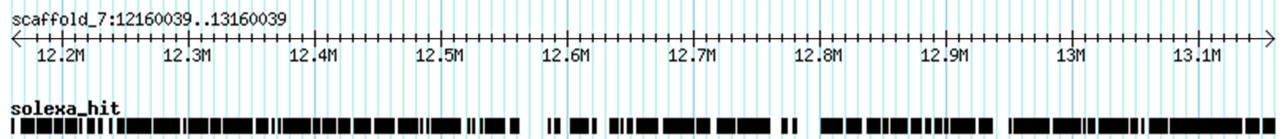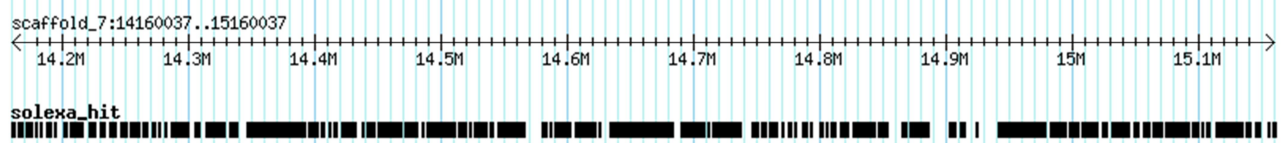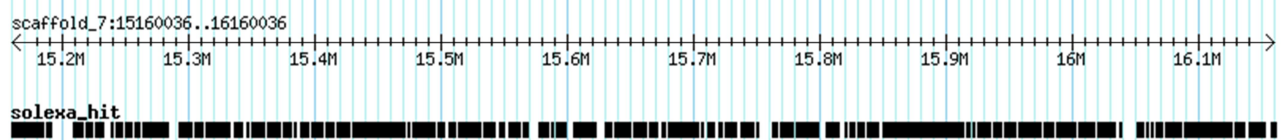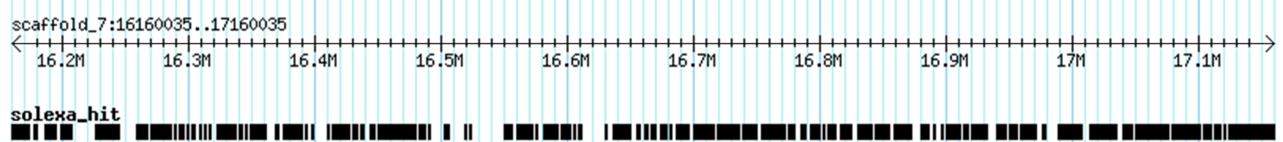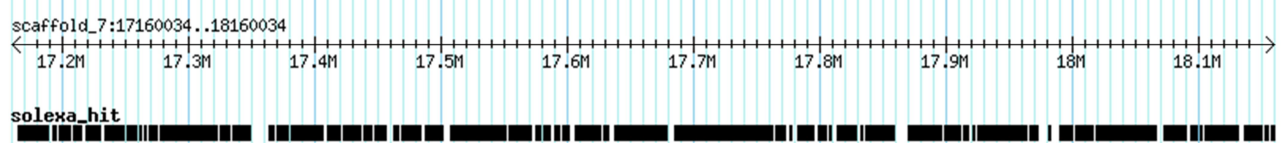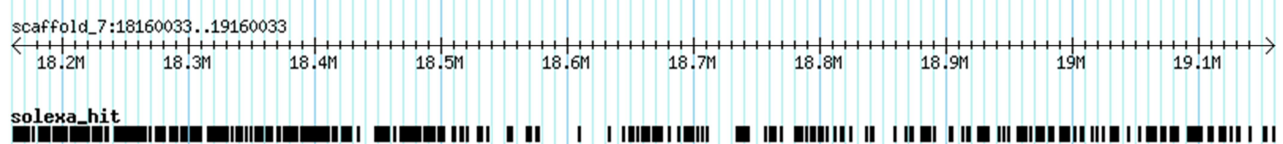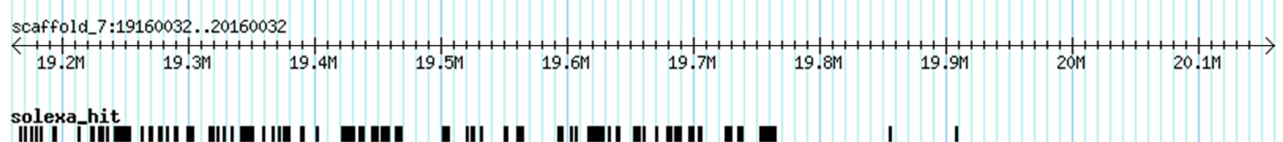

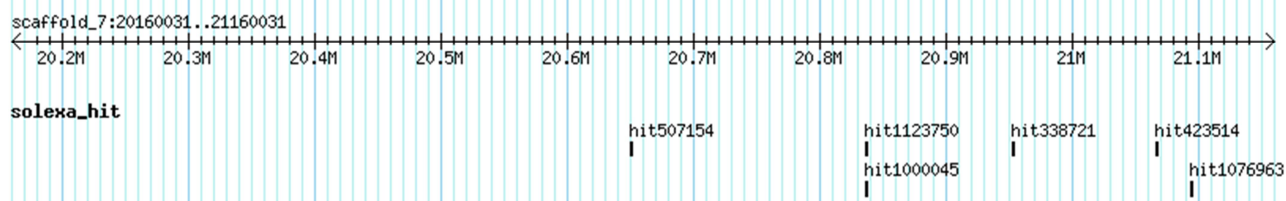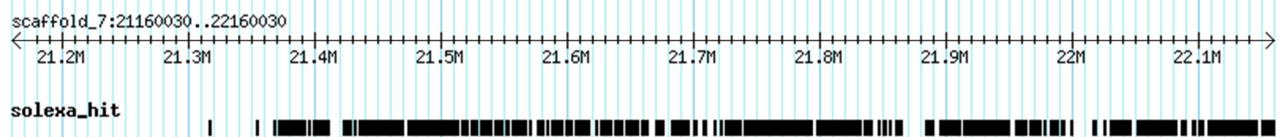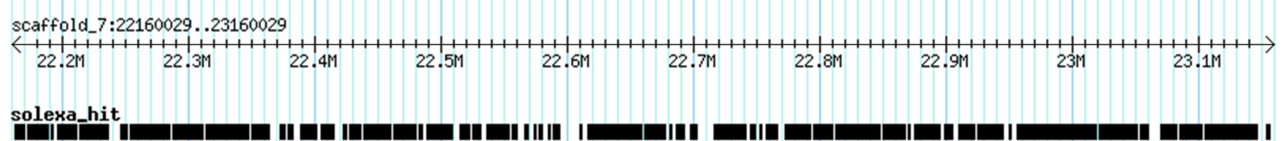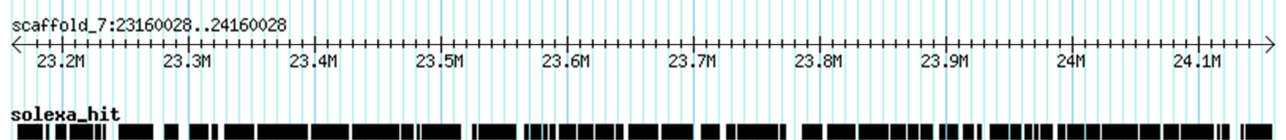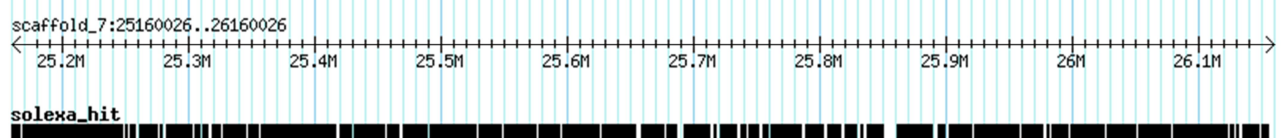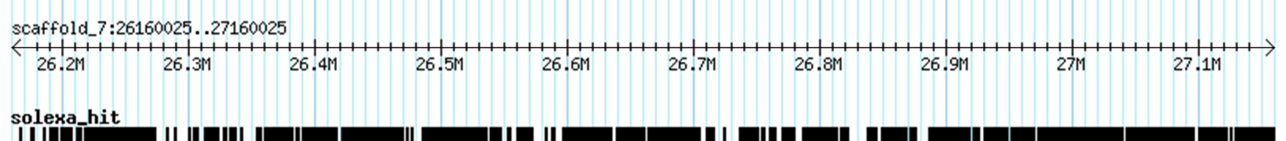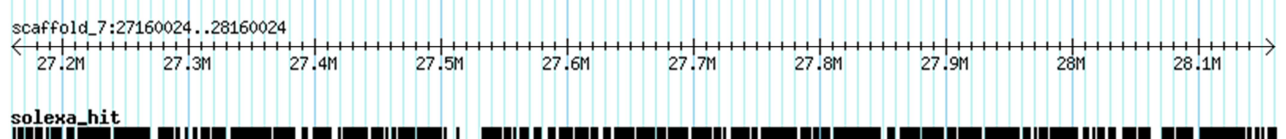

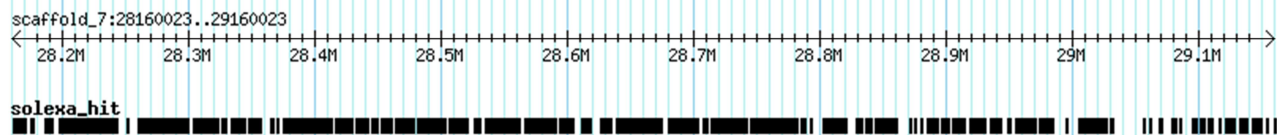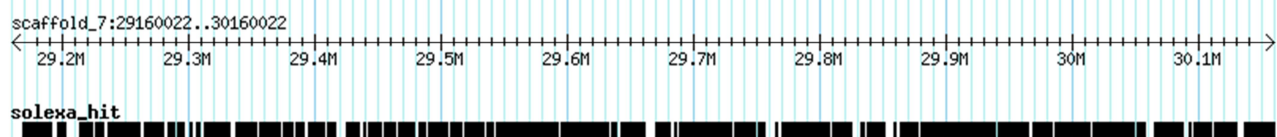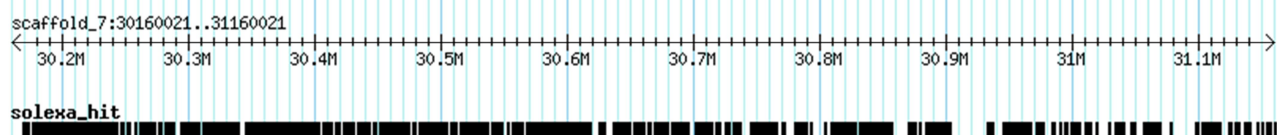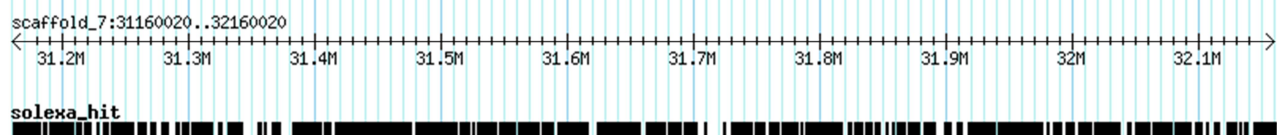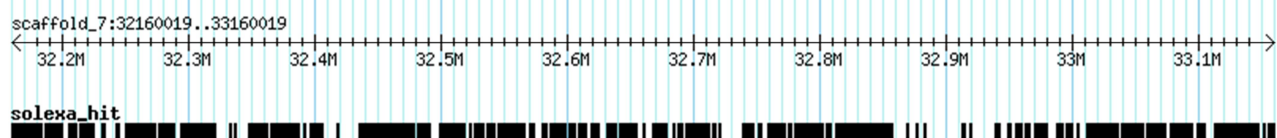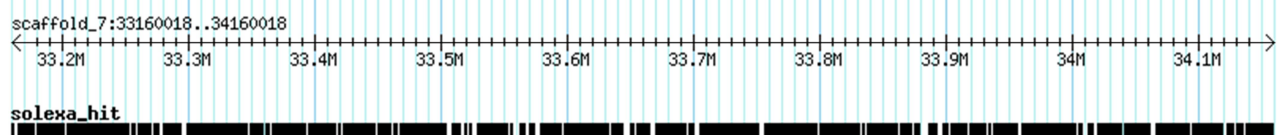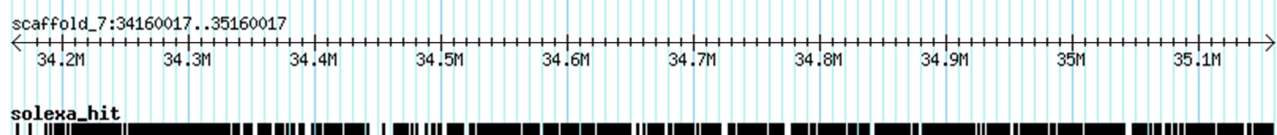

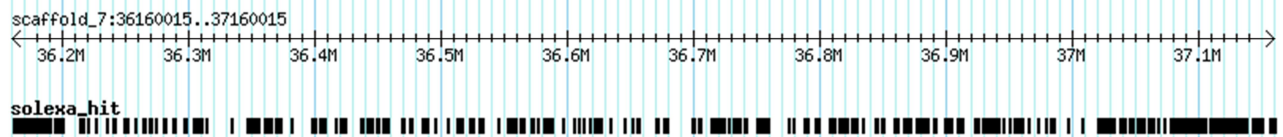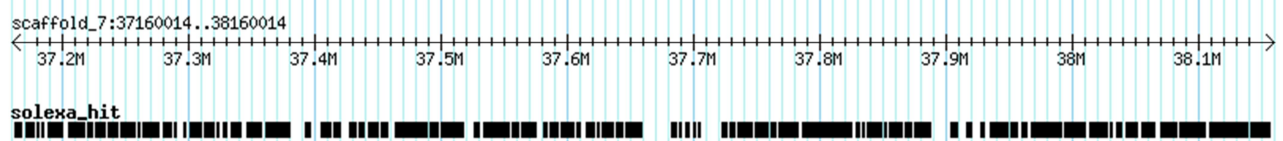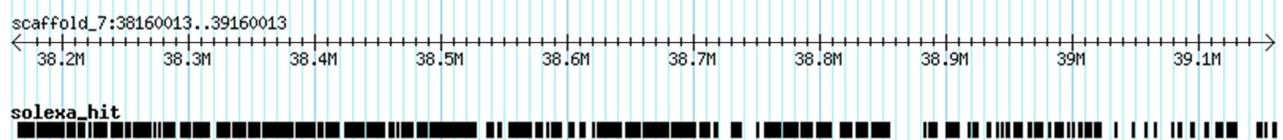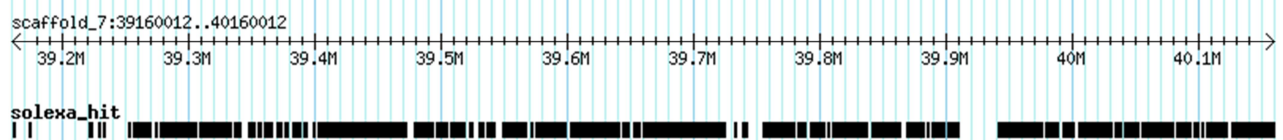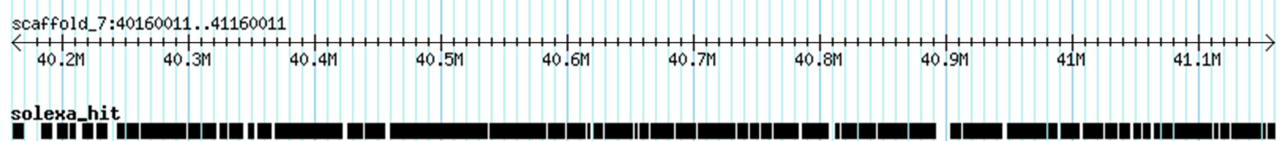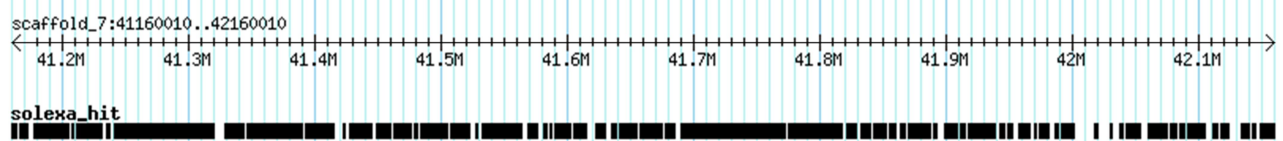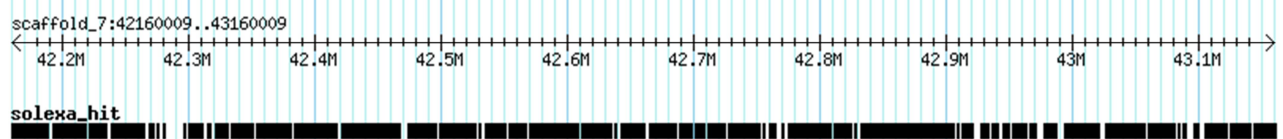

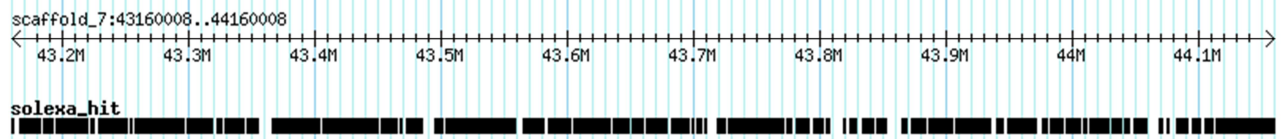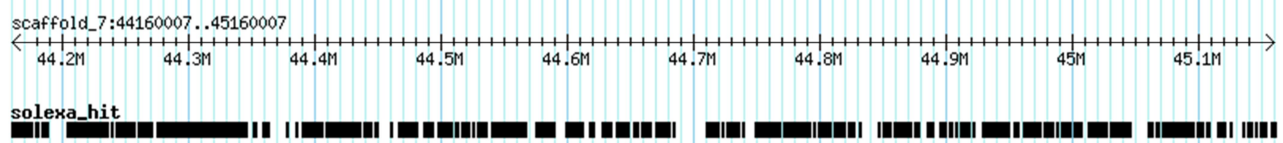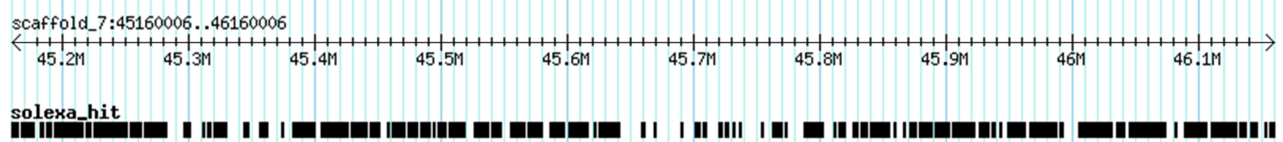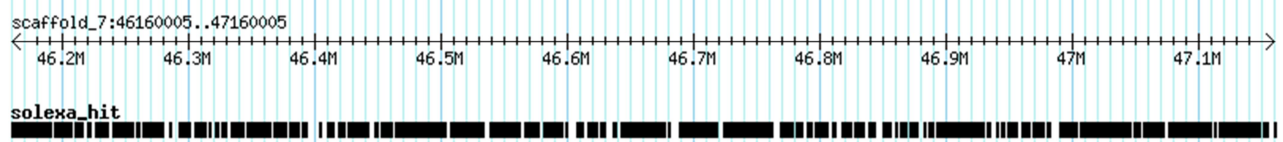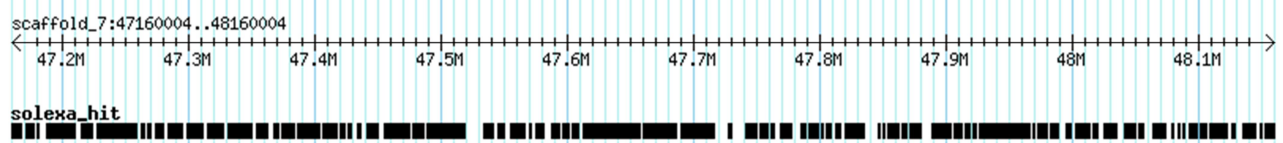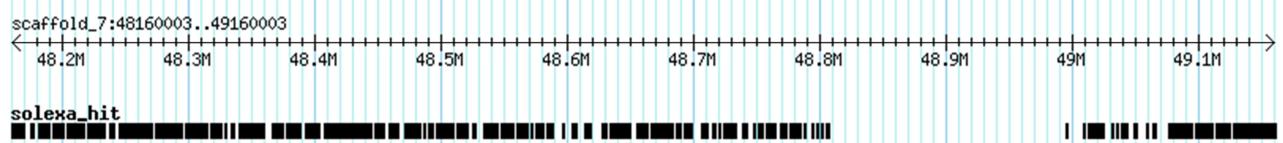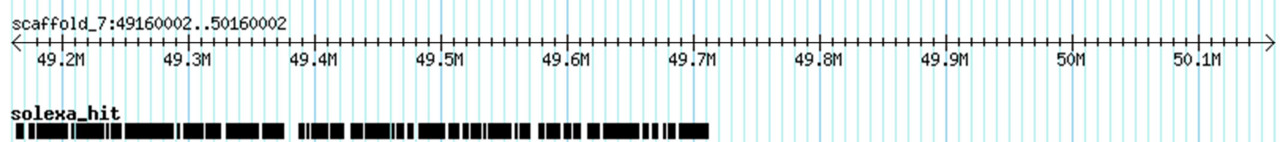

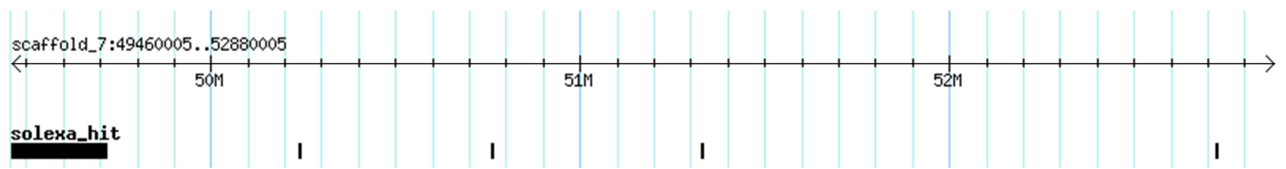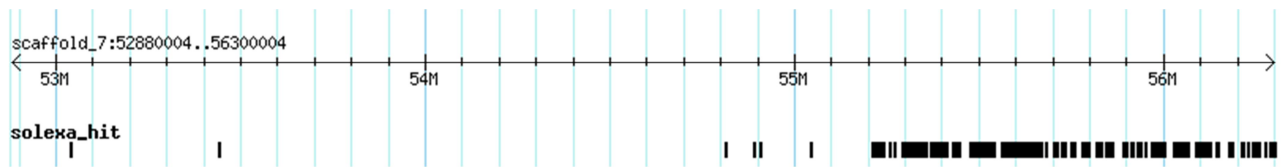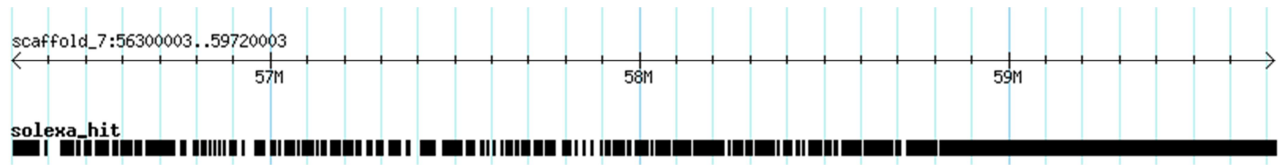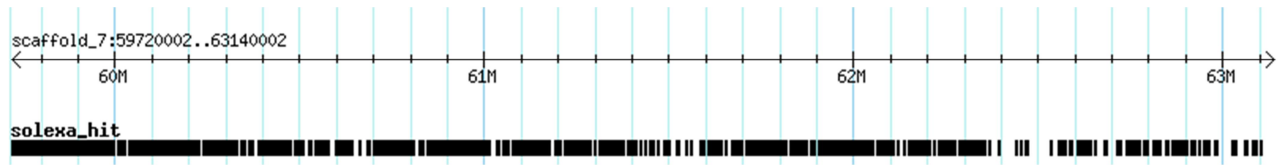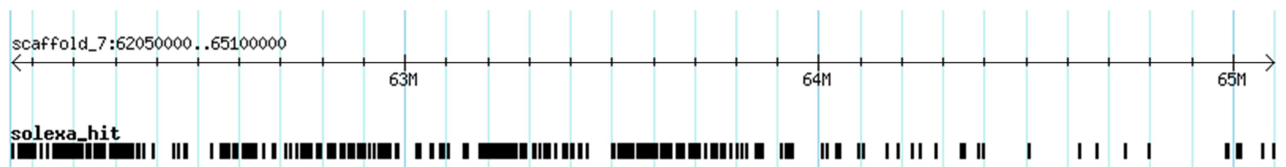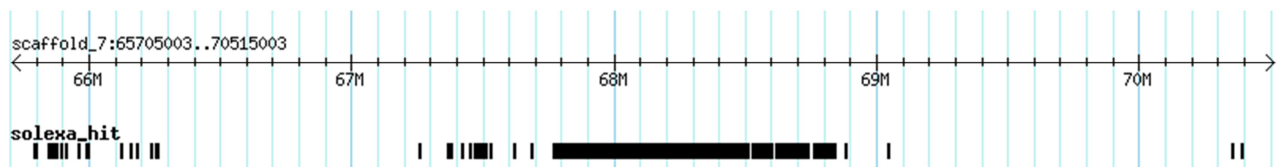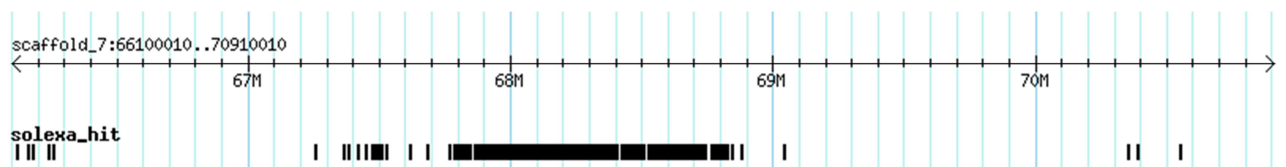

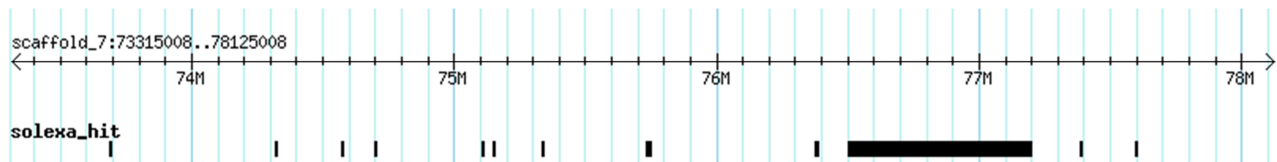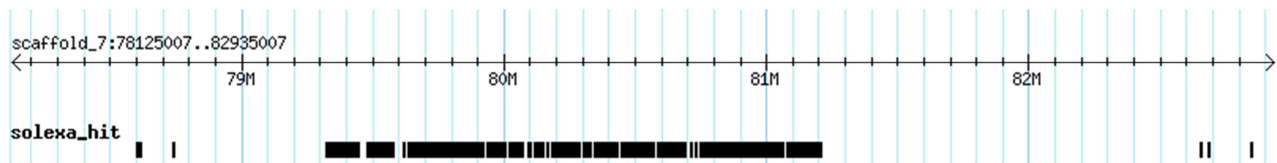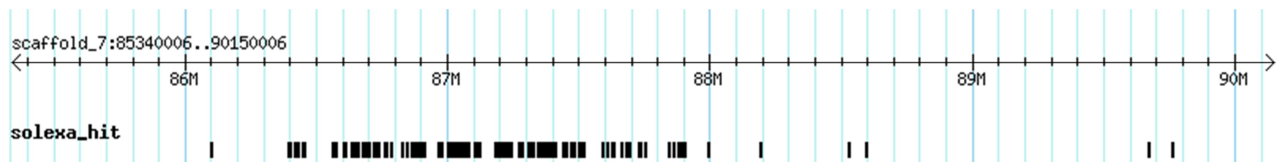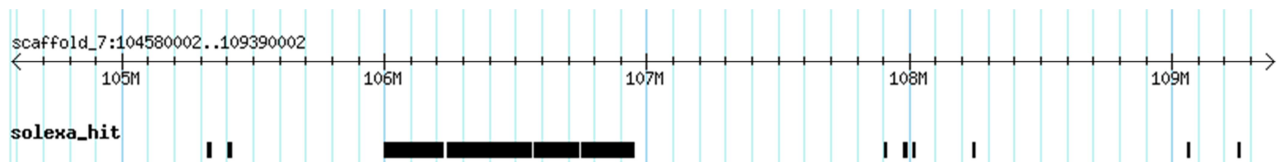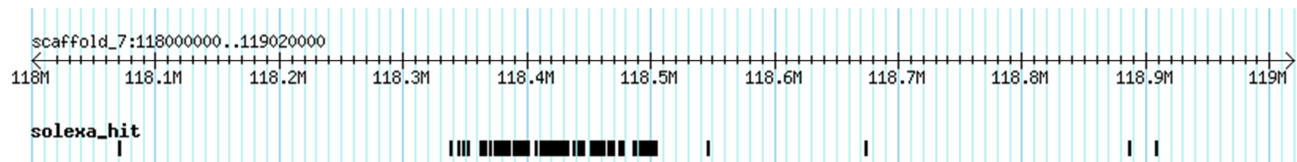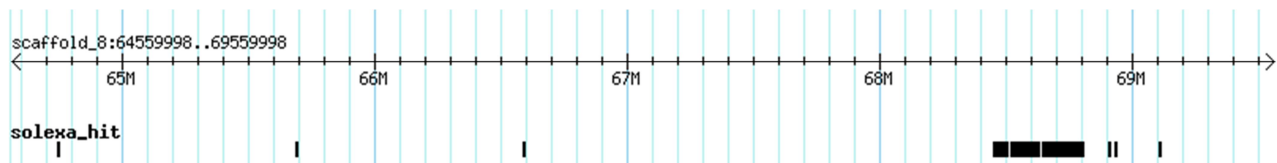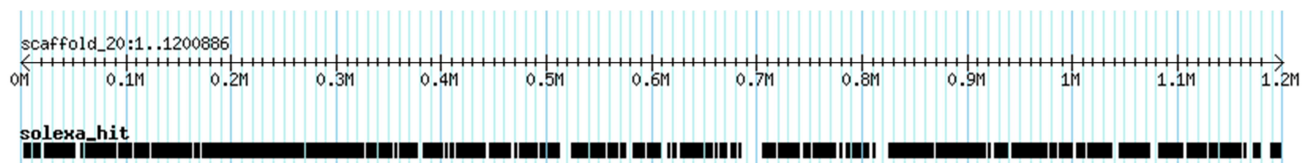

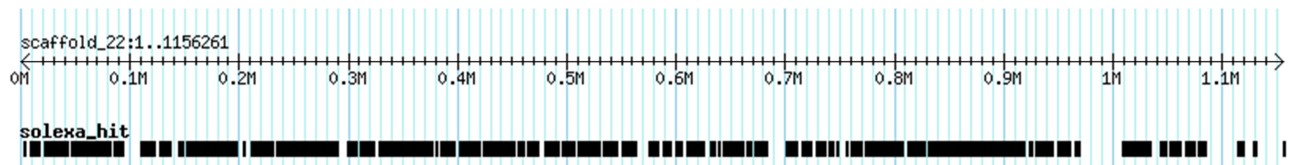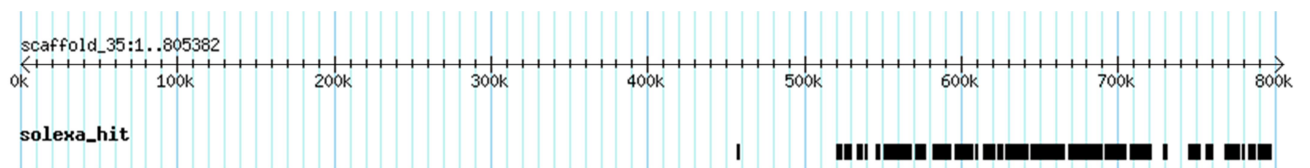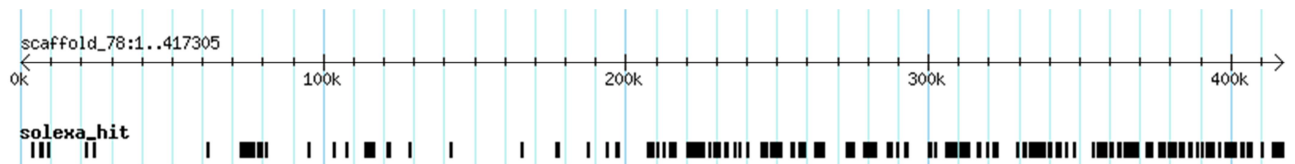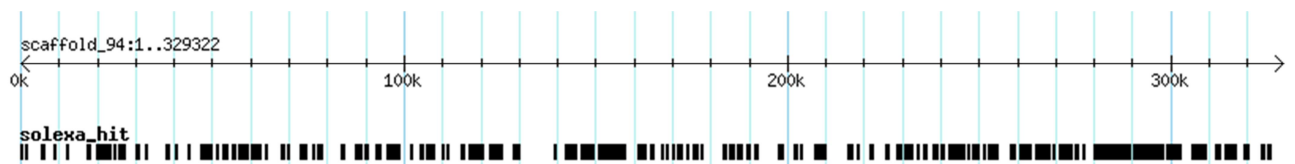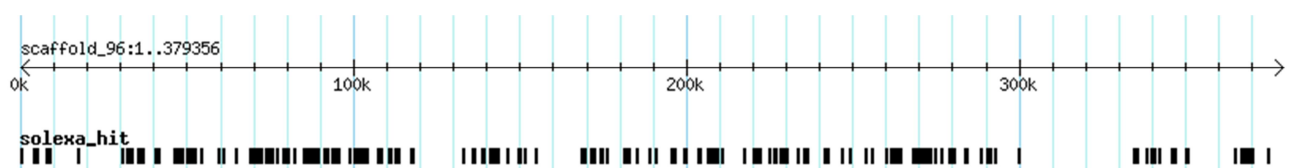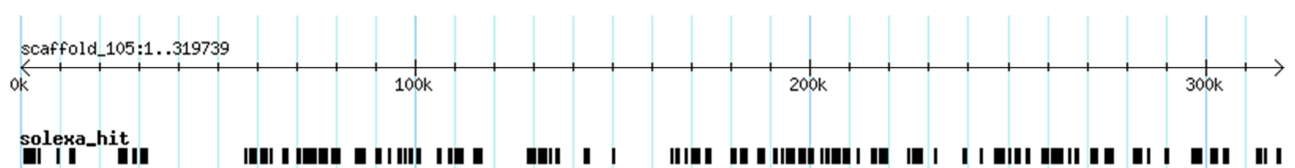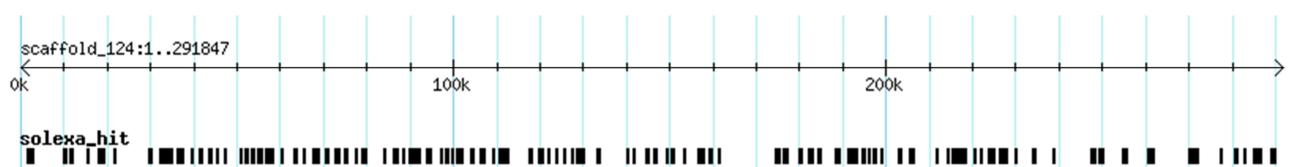

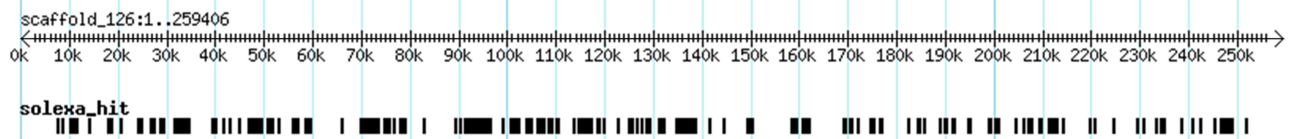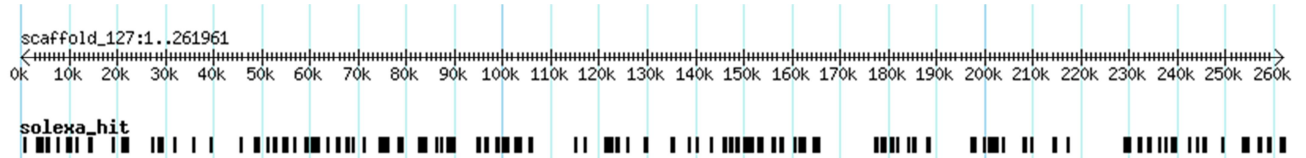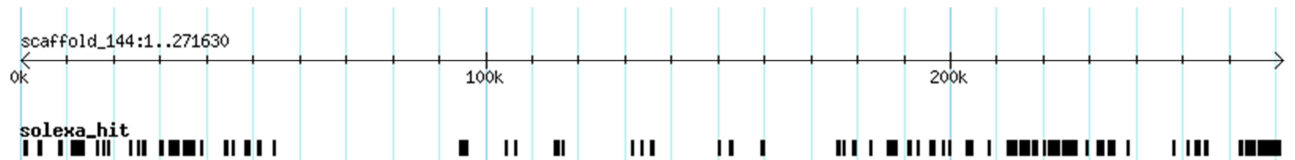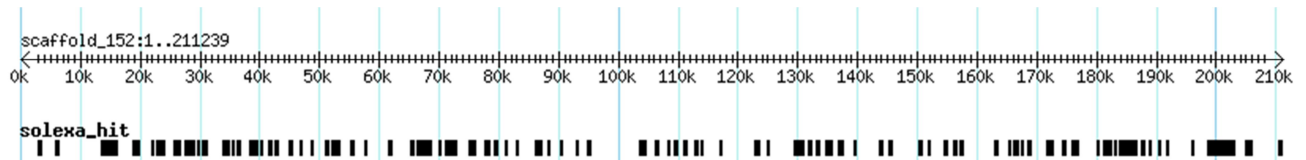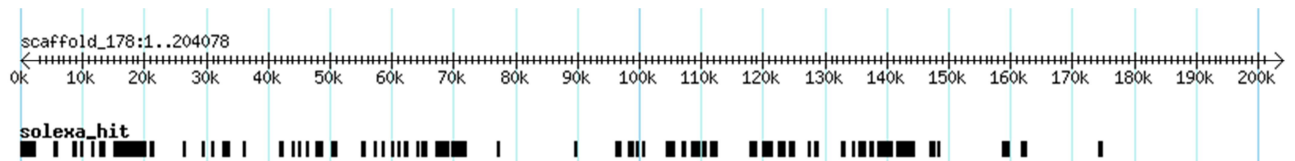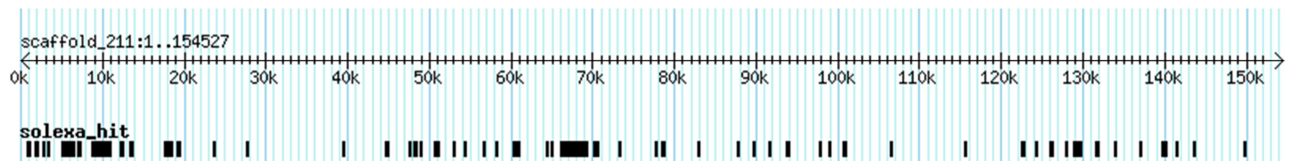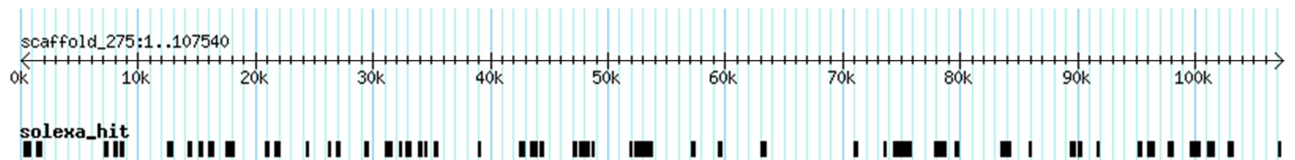

Supplement: Additional file 6 — Read distribution on v7.1 scaffolds. The file includes all scaffolds larger than 100 kb with unique reads/kb>29.23, including scaffold 35 and regions with high coverage in superscaffolds 1, 2, 3, 4 and 8. Scaffolds are ordered by ascending scaffold number. Superscaffold 7 is divided into several smaller parts. Each scaffold shows length scale and reads. [file 1471-2164-14-357-S6.pdf]
